# The Composition of Volatile Organic Compounds Correlates with the Genetic Variability Within the *Calypogeia sphagnicola* Species Complex (*Marchantiophyta*, *Calypogeiaceae*)

**DOI:** 10.3390/molecules30173642

**Published:** 2025-09-07

**Authors:** Rafał Wawrzyniak, Małgorzata Guzowska, Katarzyna Buczkowska, Alina Bączkiewicz

**Affiliations:** 1 Faculty of Chemistry, Adam Mickiewicz University, Uniwersytetu Poznańskiego 8, 61-614 Poznań, Poland; gosia.guzowska@gmail.com; 2 Faculty of Biology, Adam Mickiewicz University, Uniwersytetu Poznańskiego 6, 61-614 Poznań, Poland; alinbacz@amu.edu.pl

**Keywords:** *Calypogeia sphangicola*, liverworts, genetic markers, speciation, volatile organic compounds, HS-SPME, GC-MS

## Abstract

This paper presents the first comprehensive analysis of the composition of volatile organic compounds (VOCs) present in the liverwort *Calypogeia sphagnicola* belonging to the *Calypogeiaceae* family. Based on DNA markers, three genetically distinct groups were examined: *C. sphagnicola* f. *sphagnicola*; *C. sphagnicola* f. *paludosa*; and *C. sphagnicola* LC. The volatile organic compounds were determined using headspace solid-phase microextraction (HS-SPME) and analyzed by gas chromatography combined with mass spectrometry (GC-MS). A total of 65 organic compounds were detected from the tested plant material and 42 compounds were identified. The chemical analysis revealed distinct VOC profiles corresponding to three genetically defined groups. Sesquiterpenes (49.91–64.21%) and sesquiterpenoids (4.99–11.56%) dominated the VOC profiles, followed by monoterpenes (0.95–4.73%), aromatic compounds (2.43–5.12%), and aliphatic compounds (0.74–1.55%). It is noteworthy that aliphatic compounds were absent in *C. sphagnicola* f. *paludosa*, whereas the most abundant compounds were bicyclogermacrenes (20.92–33.60%) and anastreptenes (6.75–14.95%). Marker compounds were selected to allow for the rapid identification of individual genetic groups.

## 1. Introduction

The genus *Calypogeia* Raddi represents leafy liverworts classified under the subclass *Jungermanniidae*. Liverworts (*Marchantiophyta*), in addition to mosses (*Bryophyta*) and hornworts (*Anthocerophyta*), are one of the three divisions of plants known as bryophytes [1]. Liverworts were among the first plants to colonize land and played an important role in the evolution of early land plants [2,3,4]. Liverworts are a diverse phylum of small, herbaceous, terrestrial plants that is estimated to comprise about 7000 species [5], and new species are still being discovered [6]. Today, liverworts are an important component of many terrestrial ecosystems [7]. Liverworts are known to contain numerous biologically active compounds, such as terpenoids and aromatic compounds, which are synthesized and accumulated in oil bodies, cell structures characteristic to only these plants [8]. Most of the chemical compounds present in liverworts are specific only to this group of plants [9,10,11]. These compounds are also a source of valuable markers used to identify difficult-to-distinguish species of higher plants, including the *Salvia* genus [12], *Pinus mugo* complex [13], grape varieties [14], rice cultivars [15] and liverwort species [16,17,18]. Liverworts are also a rich source of bioactive compounds with promising medical potential. Among these are bibenzyl and bisbibenzyl compounds, which are found in species such as *Marchantia emarginata*, *Asterella angusta*, *Plagiochila sciophila*, *Dumortiera hirsute*, and *Radula marginata*. These compounds have demonstrated anticancer [19,20,21], antiviral [22], antifungal [23], and psychoactive activities [24].

Liverworts are plants that typically inhabit moist and humid environments characterized by specific microclimatic conditions [25,26]. Due to their high sensitivity to environmental changes, particularly within microhabitats, liverworts are increasingly threatened with extinction in many regions of the world [27]. Despite their ecological significance, our understanding of liverworts remains limited compared to vascular plants. Numerous taxonomic and evolutionary issues concerning this group are still unresolved.

The genus *Calypogeia* contains approximately 90 species and has a wide geographical distribution [25]. Nine species of this genus occur in Europe: *C. arguta* Nees & Mont., *C. azorica* Bischl., *C. azurea* Stotler & Crotz, *C. fissa* (L.) Raddi, *C. integristipula* Steph., *C. muelleriana* (Schiffn.) Müll. Frib., *C. neesiana* (Massal. & Carestia) Müll. Frib., *C. sphagnicola* (Arnell & J. Perss.) Warnst. & Loeske, and *C. suecica* (Arnell & J. Perss.) Müll. Frib. [5]. Recent molecular taxonomic studies of the genus *Calypogeia* have demonstrated that some taxonomically recognized species are, in fact, species complexes composed of two or three genetically distinct groups. Genetically differentiated plant groups have also been identified, including within *C. sphagnicola*, suggesting the presence of cryptic diversity within this species [28,29]. *Calypogeia sphagnicola* is considered to have a wide distribution throughout the Holarctic and is being reported from North America, Europe, and Asia [25,26,30]. The appearance of this species is closely related to the peat bogs, where the plants grow intertwined between the stems of the peat mosses.

Three genetically distinct groups within *C. sphagnicola* have been designated: *C. sphagnicola* f. *sphagnicola*, *C. sphagnicola* f. *paludosa*, and *C. sphagnicola* LC [28,29]. The groups in Poland have an allopatric pattern of geographic distribution: *C. sphagnicola* f. *sphagnicola* occurs exclusively in the lowlands of the northern part of the country on raised peat bogs, whereas *C. sphagnicola* f. *paludosa* is found only in the mountains of southern Poland, mainly in the subalpine zone, where it grows on *Sphagnum-Polytrichum hummocks* on the upper part of north-facing slopes, and *C. sphagnicola* LC occurs only at the foot of the Tatra Mountains [28,29]. Strong evidence for species separation is provided by the difference in genome size and ploidy level: *C. sphagnicola* f. *sphagnicola* is haploid, whereas *C. sphagnicola* f. *paludosa* is a diploid group [28,29]. However, due to the lack of diagnostic morphological characters, the identified groups have not yet been formally described as a species, but genetic evidence supports the hypothesis that these groups constitute separate species. The morphological differences among the groups are small and mainly refer to size, which unfortunately is not a strong diagnostic feature. *Calypogeia sphagnicola* f. *sphagnicola* are small plants, with leaves longer than wide, small, and deeply cut underleaves, with colorless, often undivided oil bodies present in all cells of the lateral leaves and underleaves and abundantly producing gemmae. *Calypogeia sphagnicola* f. *paludosa* and *C. sphagnicola* LC, on the other hand, are much larger plants with similar leaves and underleaf shape, with colorless, more divided oil bodies; these plants do not produce propagules. In such a situation, another source of evidence that confirms the differences among species may be the composition of volatile organic compounds (VOCs). As shown by Asakawa [9,10,11], liverworts contain many enantiomers of compounds that occur in higher plants and that are characteristic only of this group of plants, thus serving as markers. The usefulness of volatile organic compounds for species delimitation was demonstrated in studies on *Pellia*, *Riccardia*, *Pallavicinia*, *Mylia*, *Porella*, and *Conocephalum* [10,18,31,32,33]. Similarly, previous chemotaxonomic studies of *Calypogeia* have shown that certain species differ in their chemical compound profiles [9,10,11]. However, chemical investigations of *C. azurea*, *C. sphagnicola*, *C. integristipula*, and *C. neesiana* have focused exclusively on the isolation and characterization of single compounds [34,35]. More comprehensive chromatographic analyses of compound compositions have been conducted only for *C. muelleriana*, *C. fissa*, and *C. suecica* [36,37,38]. At the species level, the composition of VOCs has been shown to be stable and varies little depending on the location or geographical origin [9,10,11]. Much greater fluctuations were observed in plants collected at different times of the year [39,40].

The purpose of this study was to compare the VOC profiles of three genetically distinct forms of *C. sphagnicola* using GC-MS and to assess whether their chemical differences support their classification as separate taxa. To the best of our knowledge, no previous research has integrated chemical and genetic analyses to investigate the chemotaxonomic diversity within *C. sphagnicola*.

## 2. Results and Discussion

### Volatiles Present in Calypogeia sphagnicola

**Figure 1:**
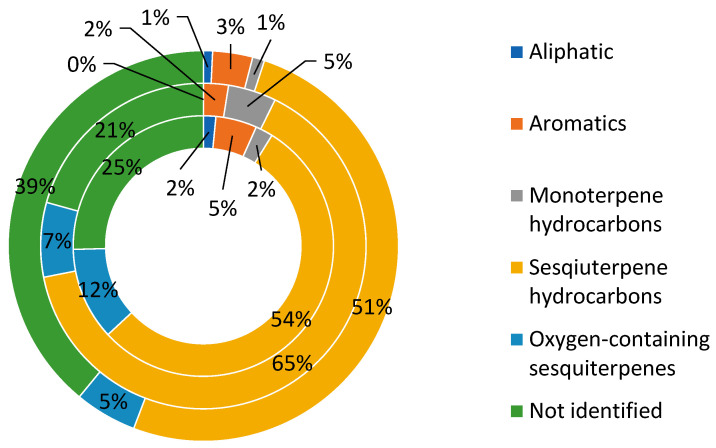
Average contents of compounds collected in years 2021–2022, divided into groups as follows: *C. sphagnicola* f. *sphagnicola* (CSS) in inner circle, *C. sphagnicola* f. *paludosa* (CSP) in middle circle, and *C. sphagnicola* (CSL) in outer circle.

The content of volatile organic compounds (VOCs) was analyzed in 38 *C. sphagnicola* samples, which were divided into three genetic groups based on DNA markers: *C. sphagnicola* f. *sphagnicola* (CSS), *C. sphagnicola* f. *paludosa* (CSP), and *C. sphagnicola* LC (CSL) (Figure S1). The study material was collected over two consecutive years from the same locations, with 19 samples collected in 2021 (Table S1a–e) and 19 in 2022 (Table S2a–e). The results presented in this study represent the first comprehensive published account of metabolite diversity in *C. sphagnicola* in relation to its genetic differentiation. Table S1a,b present the results for the *C. sphagnicola* f. *sphagnicola* samples. Table S1c,d present the results for the *C. sphagnicola* f. *paludosa* samples. Table S1e presents the results for the *C. sphagnicola* LC samples. The results of the samples collected in 2022 were grouped in a similar way. A total of 65 compounds were detected from the liverwort samples, of which 42 compounds were identified (Table S3). The unidentified compounds were described using mass spectra containing information on the basic ions, and depending on the *C. sphagnicola* group, they ranged from 21% to 39% (Figure 1). The total contents of the identified compounds for *C. sphagnicola* f. *sphagnicola*, *C. sphagnicola* f. *paludosa*, and *C. sphagnicola* LC were at a similar level to those for the *C. suecica* group 1 [41]. The lower total contents of the identified compounds in the *C. sphagnicola* LC samples resulted from the fact that the group of unidentified compounds included a dominant component with a retention index of 1694 (**57**). The average values calculated based on the values from Table S1a–e and Table S2a–e for individual *C. sphagnicola* groups divided into 2021 and 2022 are presented in Table 1. The absolute totals of the volatile peak areas per 5 mg of sample were similar for the three genetic groups of *C. sphagnicola*.

In the liverwort *C. sphagnicola* f. *sphagnicola* samples, 64 volatile organic compounds were determined. In the *C. sphagnicola* f. *paludosa* samples, 56 compounds were detected. In the *C. sphagnicola* LC samples, there were the fewest compounds, only 55.

The *C. sphagnicola* samples tested were dominated by compounds belonging to sesquiterpenes (49.91–64.21%), which are hydrocarbons, and sesquiterpenoids (4.99–11.56%), which are oxygenated derivatives of sesquiterpenes. In addition to the above-mentioned groups of compounds, the tested samples contained compounds belonging to monoterpenes (0.95–4.73%), aromatic compounds (2.43–5.12%), and aliphatic compounds (0.74–1.55%). In the case of the *C. sphagnicola* f. *paludosa* samples, no aliphatic compounds were detected (Figure 1). No significant differences in the VOC composition were observed between the samples collected in 2021 and 2022. The detailed percentage share of a given group of compounds in *C. sphagnicola*, divided into genetic groups and the year of collection of the research material, is presented in Figure S2a–c.

The group of sesquiterpenes is represented by bicyclogermacrene (**34**), which was the dominant component in all the tested samples. Its content differentiated the tested groups of *C. sphagnicola*. In the case of *C. sphagnicola* f. *sphagnicola*, this compound occurred at a level of 25.44–25.78%; in the case of *C. sphagnicola* f. *paludosa*, it occurred at a level of 33.23–33.60%; and the *C. sphagnicola* LC samples contained it at 20.92–21.48%. Moreover, in the *C. sphagnicola* LC samples, the dominant component (29.05–29.24%) was an unidentified compound with a retention index of 1694 (**57**). Based on the MS spectrum, it can be determined that it also belongs to the sesquiterpene group. The compound differentiating the discussed *C. sphagnicola* groups was anastreptene (**20**). The highest content of this compound (14.13–14.95%) was recorded for *C. sphagnicola* LC. In the case of *C. sphagnicola* f. *paludosa* and *C. sphagnicola* f. *sphagnicola*, these values were lower and amounted to 9.27–9.33% and 6.75–6.77%, respectively. The remaining compounds belonging to the sesquiterpene group also differentiated the species studied, but not to such a significant extent. These included the following: bicycloelemene (**18**), α-ylangene (**21**), β-elemene (**22**), α-gurjunene (**23**), (-)-aristolene (**25**), γ-maaliene (**26**), α-maaliene (**27**), alloaromadendrene (**28**), γ-gurjunene (**29**), germacrene D (**32**), ledene (**33**), γ-humulene (**35**), cuparene (**36**), α-bulnesene (**37**), valencene (**38**), 4,5,9,10-dehydro-isolongifolene (**39**), and 1,4-dimethyl-7-isopropyl-azulene (**58**). In the case of valencene (**38**), the presence of this compound was not recorded in *C. sphagnicola* f. *paludosa*; thus, it can be used to differentiate this genetic group from *C. sphagnicola* f. *sphagnicola* and *C. sphagnicola* LC.

Compounds belonging to the sesquiterpenoid group were present in the studied *C. sphagnicola* samples at a lower level. The dominant component of this group of compounds was ledene oxide-(II) (**49**). The highest content of this compound, 4.42–4.47%, was recorded in *C. sphagnicola* f. *paludosa*. In *C. sphagnicola* f. *sphagnicola*, the content was 2.71–2.73%, and in *C. sphagnicola* LC, it was 0.86–0.90%. In the case of other compounds belonging to sesquiterpenoids, their content in the biological material studied did not exceed 2.70%. Within this group, the following compounds were determined: spathulenol (**41**), globulol (**44**), bisabola-2,10-diene 1,9-oxide (**46**), isospathulenol (**50**), α-acorenol (**51**), aromadendrane-4,10-diol (**54**), and geranyllinalool (**63**). Also within this group of compounds, globulol (**44**) was not present in *C. sphagnicola* LC, and isospathulenol (**50**) was not present in *C. sphagnicola* f. *paludosa*.

The group of monoterpenes was represented by only six compounds: tricyclene (**4**), α-pinene (**5**), camphene (**7**), β-pinene (**9**), 3-carene (**11**), and β-cyclocitral (**15**). The content of individual compounds from this group in the tested plant material did not exceed 1.58%. It was also observed that 3-carene (**11**) occurred only in *C. sphagnicola* f. *paludosa* and β-cyclocitral (**15**) was not detected from *C. sphagnicola* LC.

Aromatic compounds in the tested liverworts occurred in amounts similar to that of the compounds from the monoterpene group. The following compounds were from this group: benzaldehyde (**8**), benzenemethanol (**12**), benzeneethanol (**14**), and phenoxyethanol (**16**).

**Figure 2:**
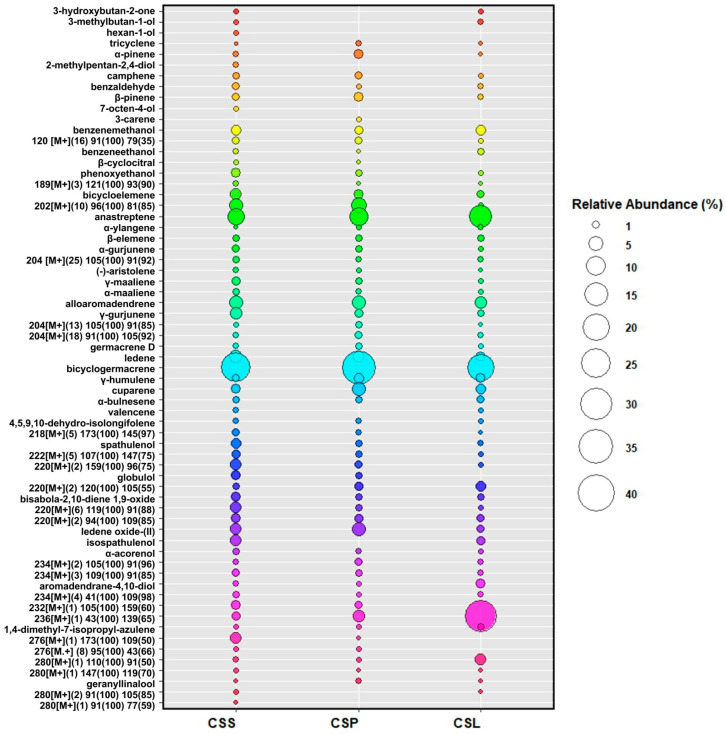
The bubble plot depicting differences in chemical compounds in genetic groups of *C. sphagnicola*: *C. sphagnicola* f. *sphagnicola* (CSS), *C. sphagnicola* f. *paludosa* (CSP), and *C. sphagnicola* LC (CSL). The genetic group is shown on the *X*-axis and the bubble size represents the concentration of a given compound.

In the case of aliphatic compounds, the following compounds were determined from *C. sphagnicola*: 3-hydroxybutan-2-one (**1**), 3-methylbutan-1-ol (**2**), hexan-1-ol (**3**), 2-methylpentan-2,4-diol (**6**), and 7-octen-4-ol (**10**). The content of individual compounds belonging to this group in the plant material did not exceed 0.54%. It turned out that, also in the case of this group, not all of the above-mentioned compounds were present in each of the genetic groups tested. Thus, hexan-1-ol (**3**), 2-methyl-2,4-pentandiol (**6**), and 7-octen-4-ol (**10**) were present only in *C. sphagnicola* f. *sphagnicola*. On the other hand, in *C. sphagnicola* f. *paludosa*, aliphatic compounds were not found; thus, they can also be used to differentiate this genetic group from other *C. sphagnicola*. The dependencies described above are shown in the bubble plot (Figure 2).

The collected results of chemotaxonomic studies within the studied samples did not show differences resulting from the habitat or the year of collection of the plant material for research, but only from genetic differences. Due to the limited availability of the studied liverwort species in natural habitats, it was not possible to examine seasonal variation in the detected VOCs within the scope of this study.

Based on the presented results for the analysis of the composition of volatile organic compounds in *C. sphagnicola*, it can be stated that bicyclogermacrene (**34**) and anastreptene (**20**) are good markers for identifying individual groups of this species. Bicyclogermacrene (**34**) is the dominant component and is present in *C. sphagnicola* f. *sphagnicola* in the amount of 25.44 to 25.78%, in *C. sphagnicola* f. *paludosa* in an amount from 33.23 to 33.60%, and in *C. sphagnicola* LC in an amount from 20.92 to 21.48%. In the case of anastreptene (**20**), the contents of this compound were as follows: *C. sphagnicola* f. *sphagnicola*, 9.27–9.33%; *C. sphagnicola* f. *paludosa*, 6.75–6.77%; and *C. sphagnicola* LC, 14.13–14.95%. Based on the content of bicyclogermacrene (**34**) and anastreptene (**20**), it is also possible to distinguish individual groups of *C. sphagnicola* from other previously described *Calypogeia* species [39,40,41]. *Calypogeia azurea* contains bicyclogermacrene (**34**) in amounts from 1.38 to 15.63% and anastreptene (**20**) from 4.87 to 33.29%. In the case of *C. suecica*, the presence of these compounds was found to be 2.05–5.95% and 4.21–8.68%, respectively. Unfortunately, based on the content of bicyclogermacrene (**34**) and anastreptene (**20**), distinguishing *C. sphagnicola* LC from *C. integristipula* is not clear because these compounds are present in *C. integristipula* in amounts of 6.99–18.09% and 15.27–25.41%, respectively. For this reason, verification should be carried out based on other components. β-Pinene (**9**) can be used for this purpose, which was present in an amount of 0.47–0.52% for *C. sphagnicola* LC, but its presence was not found in *C. integristipula.*

**Figure 3:**
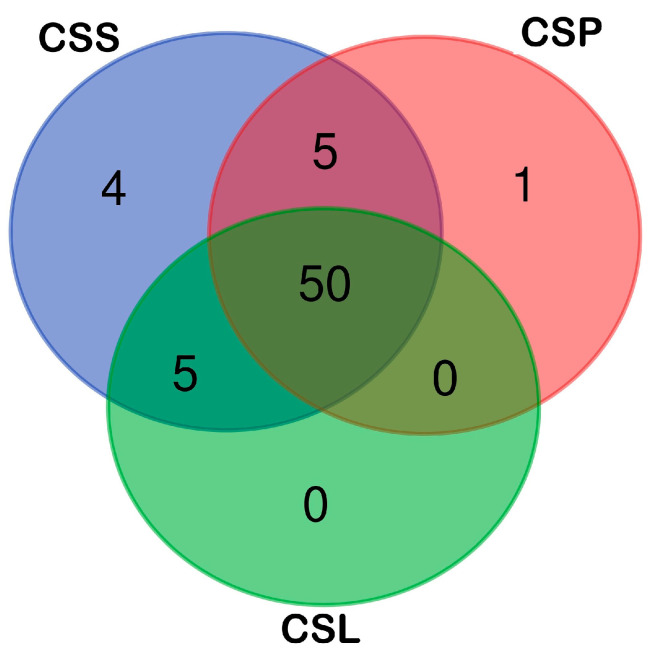
Venn diagram showing the distribution of volatile compounds in the studied groups of *C. sphagnicola*: *C. sphagnicola* f. *sphagnicola* (CSS), *C. sphagnicola* f. *paludosa* (CSP), and *C. sphagnicola* LC (CSL).

To investigate the variation in chemical compounds among the genetic groups of *C. sphagnicola*, distinguished on the basis of chloroplast DNA markers [26,27], a set of 65 detected VOCs were subjected to statistical analyses. As the Venn diagram shows (Figure 3), most of the 65 chemical compounds detected in the *C. sphagnicola* samples were common to the three groups studied. Only five volatile organic compounds (VOCs) were shared between *C. sphagnicola* f. *sphagnicola* and *C. sphagnicola* f. *paludosa* (**15**), (**44**), (**56**), (**59**), (**60**) and *C. sphagnicola* f. *sphagnicola* and *C. sphagnicola* LC (**1**), (**2**), (**38**), (**50**), (**64**). The most distinct was the *C. sphagnicola* f. *sphagnicola* group, which had four volatile organic compounds specific only to this group: 7-octen-4-ol (**10**), 2-methyl-2,4-pentandiol (**6**), 1-hexanol (**3**), and 280[M+](1) 91(100) 77(59) (**65**). *C. sphagnicola* f. *paludosa* had only one—3-carene (**11**)—while *C. sphagnicola* LC had no specific compounds. The distribution of shared and group-specific VOCs is shown in Figure 3.

**Figure 4:**
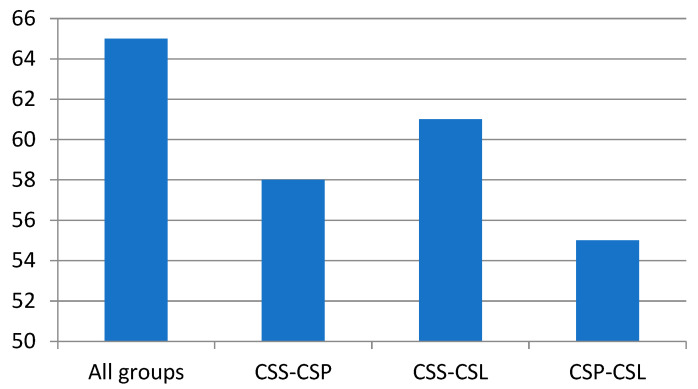
The number of compounds that differ statistically significantly across all the groups and individual group pairs of *C. sphagnicola*: *C. sphagnicola* f. *sphagnicola* (CSS), *C. sphagnicola* f. *paludosa* (CSP), and *C. sphagnicola* LC (CSL), based on an ANOVA followed by a post hoc Scheffe test (see Table S4).

A one-way analysis of variance (ANOVA) showed that the studied groups of *C. sphagnicola* differed significantly with respect to the mean of all the detected compounds shown in Table 1. The post hoc Scheffe test showed the most significant differences between the *C. sphagnicola* f. *sphagnicola* and *C. sphagnicola* LC pair, for which 61 compounds showed statistically significant (*p* < 0.05) differences, while the smallest were shown for the *C. sphagnicola* f. *paludosa* and *C. sphagnicola* LC pair, which differed statistically significantly in the mean value of 55 compounds (Figure 4, Table S4).

**Figure 5:**
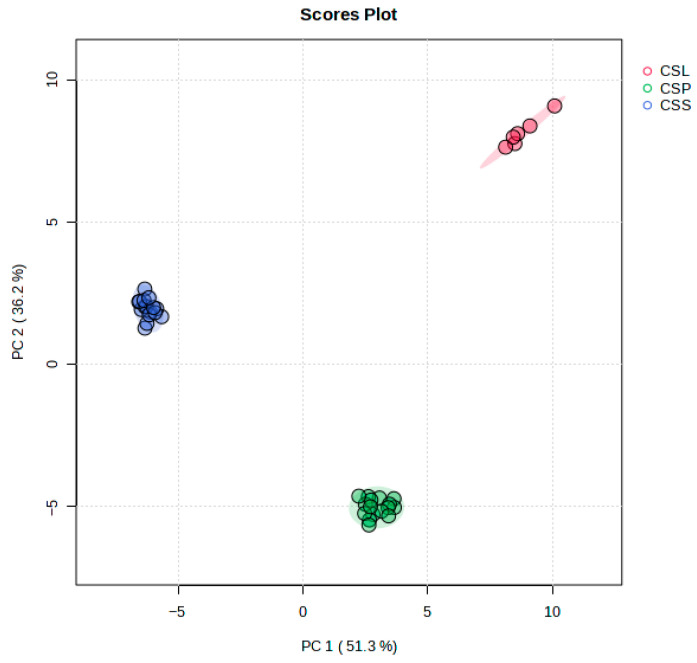
Two-dimensional PCA scatter plot based on all the detected VOCs in the *C. sphagnicola* samples: *C. sphagnicola* f. *sphagnicola* (CSS), *C. sphagnicola* f. *paludosa* (CSP), and *C. sphagnicola* LC (CSL). The percentage of explained variance (R2X) was 51.3% for PC1 and 36.2% for PC2, and the predictive ability (Q2) was 43.0% and 64.0%, respectively. Different colors indicate the genetic group affiliation. Shaded areas indicate the 95% confidence regions.

A multivariate PCA analysis revealed the presence of three subsets. The explanatory and predictive abilities of the PCA model were evaluated based on two parameters: R2X and Q2. The model obtained for the *C. sphagnicola* samples included four statistically significant components that explained 89.9% of the variation (R2X) and 83.9% of the predicted ability (Q2). However, only the first two principal components, PC1 and PC2, explained as much as 87.5% of the total variance (R2X), at 51.3% and 36.2%, respectively (Figure 5). The scatter plot of the first two principal components showed the strong distinctiveness of each of the three groups analyzed, which fully matched the groupings established on the basis of genetic studies (Figure 5).

The largest contributions to the PC1 axis were made by compounds **3**, **10**, **29**, **41**, **43**, **47**, and **59**, which had high negative (>−0.95) factor loadings. The greatest contributions to the PC2 axis were made by compounds **2**, **9**, **19**, **34**, **49**, **54**, and **61**, with high factor loadings (>0.90), where **2**, **54**, and **61** had positive factor loadings and the remaining compounds had negative factor loadings (Figure S3a–b). The above compounds had the greatest influence on the separation of the *C. sphagnicola* groups in the first two principal components. The *C. sphagnicola* f. *sphagnicola* group located on the left (negative) side of the PCA diagram, along the PC1 axis, was characterized by a higher concentration of VOCs **3**, **10**, **29**, **41**, **43**, **47**, and **59** compared to the *C. sphagnicola* f. *paludosa* and *C. sphagnicola* LC groups located on the right (positive) side of the plot. In turn, the *C. sphagnicola* LC group, which was located in the negative part of the diagram along the PC2 axis, had higher values for the **2**, **54**, **57**, and **61** VOCs and lower values for the **9**, **19**, **34**, and **49** VOCs (Figure 5). We did not observe any significant differences between samples from different locations or between subsequent years of sample collection (Figures S4 and S5).

Based on the variable importance in projection (VIP) obtained from the PLS-DA model, the chemical compounds that exhibited differences between the three genetic groups of *C. sphagnicola* were initially screened. The top 20 VOCs were selected for which the VIP values were >1 (Figure S6). The higher the VIP result, the greater the contribution of the chemical compound to group separation. Among the VOCs indicated as the most important for distinguishing the studied groups, the *C. sphagnicola* f. *sphagnicola* group was characterized by an increased content of 16 compounds and a reduced content of 4 compounds compared to the *C. sphagnicola* LC group, while the *C. sphagnicola* f. *paludosa* group showed intermediate values on most of these compounds (Figure S6). Most of the features selected based on VIP also had the highest factor loadings in PCA, and thus the greatest contribution to separating the samples into groups.

**Figure 6:**
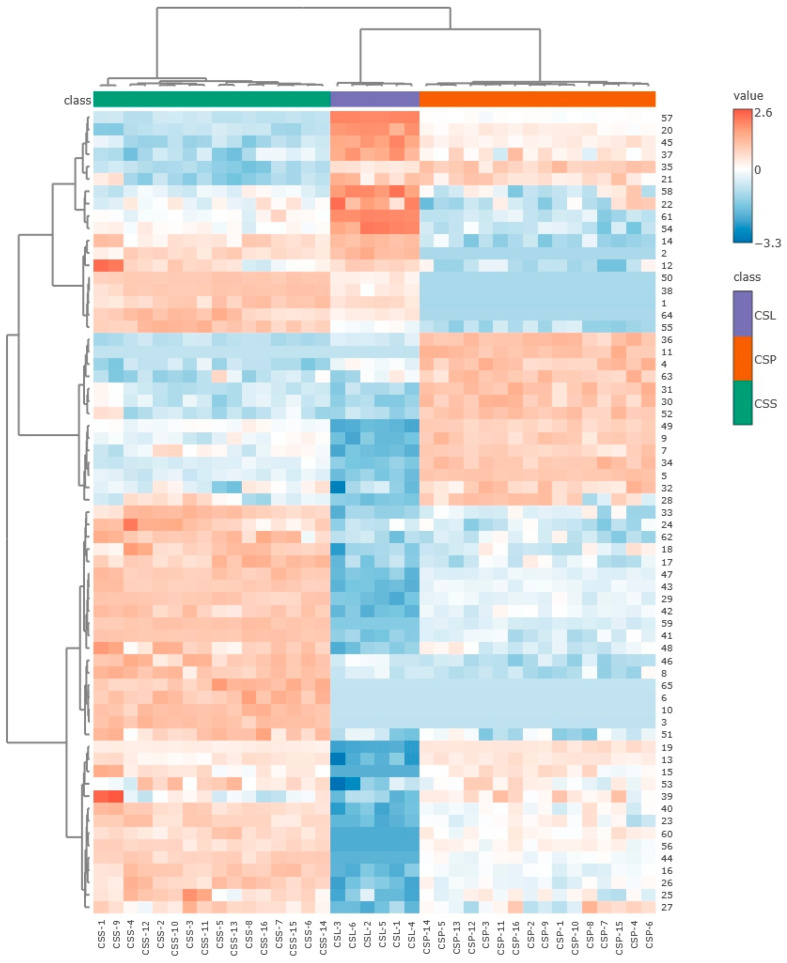
Heatmap clustering of VOC profiles from *C. sphagnicola* samples: *C. sphagnicola* f. *sphagnicola* (CSS), *C. sphagnicola* f. *paludosa* (CSP), and *C. sphagnicola* LC (CSL). The annotation bar shows the clustering of the samples by group (class). Each cell was colored based on the level of the concentration of the chemical compound in the sample.

The differentiation of the analyzed samples according to the genetic group is clearly illustrated by the heatmap. The analysis showed that both the relative content and the presence of unique volatile organic compounds (VOCs) significantly influenced the clustering of the samples. The detected compounds formed three distinct groups, whose content in the tested plants was correlated with genetic groups identified using molecular markers: *C. sphagnicola* f. *sphagnicola*, *C. sphagnicola* f. *paludosa*, and *C. sphagnicola* LC [26,27]. The *C. sphagnicola* f. *sphagnicola* group originating from the raised peat bogs in the lowlands of northern Poland differed clearly from the *C. sphagnicola* f. *paludosa* and *C. sphagnicola* LC groups. Slightly smaller differences in the VOC content occurred between the *C. sphagnicola* f. *paludosa* and *C. sphagnicola* LC groups, which come from a similar habitat, i.e., peat moss tussocks located in the foothills and mountains. In the *C. sphagnicola* f. *sphagnicola* group, the levels of compounds **33**, **24**, **62**, **18**, **17**, **47**, **43**, **29**, **42**, **59**, **41**, **48**, **46**, **8**, **65**, **6**, **10**, **3**, and **51** were higher than in the other two groups. In contrast, the *C. sphagnicola* LC group showed elevated levels of compounds **57**, **20**, **45**, **37**, **58**, **22**, **61**, and **54**. The content of compounds **36**, **11**, **4**, **63**, **31**, **30**, **52**, **49**, **9**, **7**, **34**, and **32** was higher in the *C. sphagnicola* f. *paludosa* group (Figure 6).

As demonstrated in numerous previous studies [9,10,11,31], the composition of organic compounds in liverworts can serve as a valuable source of species-specific markers. However, most chemotaxonomic studies of liverworts conducted to date have been based on single specimens, which has limited the ability to assess the intraspecific variability. Studies based on larger sample sizes have revealed intraspecific variability in the content of chemical compounds, as exemplified by *Radula marginata*, where differences in the relative proportions of bibenzyl cannabinoids were observed between chemotypes associated with different geographic locations. Additionally, seasonal variation was detected, suggesting a potential influence of environmental conditions on bibenzyl cannabinoid metabolism [24]. Similarly, intraspecific variation in chemical profiles has been demonstrated in *R. complanata*, where 39% of the variability was attributed to the tree species on which the liverworts were growing, and 25% to environmental conditions [42]. The integration of volatile organic compound (VOC) profiling with genetic analyses in liverworts has shown that variability in chemical compound profiles is primarily the result of genetic differentiation, as observed in cryptic species of *Conocephalum conicum* and *Aneura pinguis* [33,43].

This pattern is also evident in species of the genus *Calypogeia* studied to date. In *C. suecica* and *C. sphagnicola*, genetic groups identified through molecular analyses are consistently associated with specific chemical compounds that can be considered marker compounds, such as bicyclogermacrene (**34**) and anastreptene (**20**). A study involving numerous genetically characterized samples of *Calypogeia*, collected across various locations and seasons, demonstrated that environmental and geographic factors exert a comparatively minor influence on the variability of organic compounds, with genetic differences playing a more dominant role [40,41]. Nevertheless, it has been shown that the content of chemical compounds in *Calypogeia* may vary depending on the growing season, the storage conditions, and whether the plants were collected directly from their natural habitat or derived from an in vitro culture [39,40]. Therefore, for comparative studies between species, samples should be collected during the same growing season. Our previous research has indicated that the optimal period for collecting liverwort material is in late summer to autumn (August and September), due to the favorable developmental phase and optimal plant conditions resulting from the prevailing weather conditions, such as a higher humidity and lower temperatures [41].

## 3. Materials and Methods

### 3.1. Plant Material

Samples of *C. sphagnicola* were collected from natural habitats in Poland. *Calypogeia sphagnicola* f. *sphagnicola* samples were collected from sites located in northern Poland, from raised bogs. *Calypogeia sphagnicola* f. *paludosa* and *C. sphagnicola* LC samples were collected from sites located in southern Poland, from peat-moss tussocks covering the mountain slopes. *Calypogeia sphagnicola* LC is a liverwort that occurs in a very limited area of the Capowski Forest, which lies at the foot of the Tatra Mountains. At the time of the publication of this article, no other sites of this liverwort could be identified. Samples with a diameter of about 5–7 cm were collected for this study. Detailed information on the location of the samples and the date of collection of the plant material is provided in Tables S5–S10.

Due to the small number of sites and the specificity of the habitat in which the studied species occurs, it was decided to collect samples only once a year, in summer and early autumn. This harvest period ensures that botanical material is obtained in the optimal development phase and the best condition of liverwort plants due to the prevailing weather conditions (a higher humidity and lower temperatures). For this study, individuals with well-developed stems and that were in a sterile state, i.e., without reproductive structures, were selected. The research materials were collected in the Warmia, Pomerania, and Tatra Mountains.

The collected samples were identified based on morphological features, as well as the structure and distribution of oil bodies in the leaves and underleaves. Finally, the samples were divided into three groups, *C. sphagnicola* f. *sphagnicola* (CSS), *C. sphagnicola* f. *paludosa* (CSP), and *C. sphagnicola* LC (CSL), based on the chloroplast DNA markers *trnL*, *trnG*, *trnH-psbA*, and *rpoC*1 according to Buczkowska et al. [28,29]. Detailed information on the GenBank accession numbers of the reference sequences for the analyzed DNA regions, as well as the voucher codes of the herbarium specimens from which they were derived, is available in our previously published studies on the genetic structure of *C. sphagnicola* [28,29]. Several stems with a total mass of about 15 mg were taken from each sample. Only green plants that did not show signs of drying and were not affected by visible diseases were selected for further studies. The plant material was carefully cleaned, rinsed with distilled water, and gently dried on tissue paper to remove excess water. Subsequent analyses were performed on living specimens to ensure the preservation of oil bodies and chemical compounds present in them, as the excessive drying of the plant material leads to the destruction of these organelles.

### 3.2. HS-SPME Extraction

The VOCs from *C. sphagnicola* were extracted using the headspace solid-phase microextraction technique (HS-SPME). Fused silica fibers coated with divinylbenzene/carboxen/polydimethylsiloxane (DVB/CAR/PDMS) (Merck KGaA, Darmstadt, Germany) were employed. The fibers, 2 cm in length and covered with a 50 µm DVB layer and a 30 µm CAR/PDMS layer, were conditioned for 1 h at 270 °C according to the supplier′s guidelines. A sample of 5 mg of clean plant material was placed in a 1.7 mL vial, which was hermetically sealed with a Teflon/silicone septum with an assembled magnetic cap (Lab Logistic Group GmbH, Meckenheim, Germany) and heated to 50 °C. The extraction of the compounds was conducted at 50 °C for 60 min. The desorption of the analytes from the fibers was performed in the injection port of the gas chromatograph at 250 °C for 10 min. The sorption and desorption operations were performed using the TriPlus RSH autosampler equipped with an SPME tool and agitator (Thermo Scientific, Waltham, MA, USA).

### 3.3. GC-MS Analysis

Volatile organic compounds (VOCs) underwent an analysis with gas chromatography–mass spectrometry (GC-MS) following the protocol detailed in reference [42]. A Trace 1310 GC system (Thermo Scientific, Waltham, MA, USA) fitted with a Quadrex 007-5 MS capillary column (30 m × 0.25 mm i.d., 0.25 µm film; Quadrex Corporation, Bethany, CT, USA) was coupled to an ISQ QD mass spectrometer (Thermo Scientific, Waltham, MA, USA). The MS operated in electron-ionization mode at 70 eV, with a scanning m/z from 30 to 550. Helium served as the carrier gas (1.0 mL min^−1^). The oven was programmed from 60 °C to 230 °C at 4 °C min^−1^, then held isothermally at 230 °C for 40 min. The injector and transfer-line temperatures were both set to 250 °C, and injections were made in splitless mode.

The compound identities were verified by matching the EI mass spectra with database entries (NIST 2011 [44], NIST Chemistry WebBook [45], Adams 4 Library [46], and Pherobase [47]) and with literature data. The compound identities were also verified by the injection of reference standards (Table S3). The retention indices, calculated against a C7–C30 n-alkane series (Merck KGaA, Darmstadt, Germany), were also compared with published values. Quantification was based on the relative peak areas in the total ion chromatogram (TIC). Each *C. sphagnicola* sample was analyzed in triplicate to ensure reproducibility.

### 3.4. Statistical Analysis

To check whether the detected chemical compounds differentiated the analyzed groups of *C. sphagnicola* that were distinguished on the basis of molecular markers, statistical analyses were performed. The statistical significance of the differences in the VOCs between the studied groups was assessed using a one-way analysis of variance (ANOVA). A Venn diagram was used to plot the common and species-specific VOCs of each *C. sphagnicloa* group [48]. A multivariate data analysis, including a principal component analysis (PCA) and a hierarchical cluster analysis (HCA), was used to extract and display the hidden structure in the analyzed data set [49,50]. Then, we selected the 20 most important variables that differentiated the analyzed genetic groups of *C. sphagnicola* based on the variable importance in projection (VIP) value using a PLS-DA analysis [51]. To display the concentration of detected compounds across the studied samples belonging to three genetic groups of *C. sphagnicola*, we used a heatmap, which allowed for the grouping of variables (compounds) and samples simultaneously. In heatmaps, the data are displayed in a grid where each row represents a chemical compound and each column represents an examined sample. The color and intensity of the boxes indicate the concentration of a given compound. Recently, heatmaps have become a frequently used technique in biology; they are useful for visualizing high-dimensionality data [52]. The principal component analysis (PCA), partial least squares discriminant analysis (PLS-DA), and heatmap were performed using the MetaboAnalyst 6.0 web portal (https://www.metaboanalyst.ca, accessed on 16 May 2025) [53]. R package ggplot2 (R version 4.4.1)was applied to build the bubble plot. STATISTICA 13.3 (StatSoft, Poland) was used to perform the remaining analysis. Before the statistical analyses, the obtained chromatographic data were subjected to a log transformation (base 10) and auto-scaling (mean-centered and divided by the standard deviation of each variable).

## 4. Conclusions

Sesquiterpenes and sesquiterpenoids were the dominant volatile compounds across all samples. Among them, bicyclogermacrene and anastreptene showed distinct distribution patterns that allowed for discrimination between the three genetic groups. Multivariate statistical analyses (PCA and PLS-DA) and heatmaps confirmed that the groups detected based on the composition and concentration of volatile organic compounds (VOCs) in the studied *C. sphagnicola* samples were consistent with the groups determined based on genetic markers, i.e., *C. sphagnicola* f. *sphagnicola*, *C. sphagnicola* f. *paludosa*, and *C. sphagnicola* LC. The analysis also indicated that the composition of metabolites was not dependent on habitat or the year of collection of the plant material for the study. The analysis of the chemical composition of VOCs also allowed for easy distinction of the species *C. sphagnicola* discussed in this publication from other species of the genus *Calypogeia*.

The results collected in this paper will allow for the preparation of a chemotaxonomic diagram to facilitate the identification of individual species within the *Calypogeia* genus.

## Figures and Tables

**Table 1:** Mean %* and standard deviation of volatile compounds detected in *C. sphagnicola* f. *sphagnicola* (CSS), *C. sphagnicola* f. *paludosa* (CSP), and *C. sphagnicola* LC (CSL) samples.

No.	Compounds	RI ^a^	Mean of Group					
			CSS		CSP		CSL	
			2021	2022	2021	2022	2021	2022
1	3-hydroxybutan-2-one	<700	0.32 (0.02)	0.30 (0.02)	-	-	0.23 (0.01)	0.23 (0.01)
2	3-methylbutan-1-ol	706	0.25 (0.02)	0.21 (0.01)	-	-	0.54 (0.02)	0.51 (0.03)
3	hexan-1-ol	867	0.28 (0.03)	0.30 (0.02)	-	-	-	-
4	tricyclene	926	0.05 (0.01)	0.06 (0.01)	0.34 (0.03)	0.35 (0.02)	0.11 (0.01)	0.12 (0.01)
5	α-pinene	936	0.39 (0.03)	0.36 (0.02)	1.56 (0.05)	1.54 (0.05)	0.14 (0.01)	0.12 (0.01)
6	2-methylpentan-2,4-diol	938	0.41 (0.03)	0.38 (0.02)	-	-	-	-
7	camphene	953	0.56 (0.04)	0.56 (0.03)	0.85 (0.04)	0.84 (0.03)	0.23 (0.01)	0.24 (0.01)
8	benzaldehyde	960	1.01 (0.05)	1.05 (0.04)	0.31 (0.02)	0.28 (0.02)	0.32 (0.02)	0.38 (0.02)
9	β-pinene	978	0.97 (0.05)	1.00 (0.04)	1.58 (0.06)	1.58 (0.05)	0.52 (0.03)	0.47 (0.03)
10	7-octen-4-ol	982	0.30 (0.02)	0.32 (0.02)	-	-	-	-
11	3-carene	1009	-	-	0.24 (0.01)	0.28 (0.02)	-	-
12	benzenemethanol	1023	1.95 (0.08)	1.98 (0.06)	1.30 (0.05)	1.30 (0.06)	2.02 (0.05)	2.04 (0.06)
13	120[M+](16) 91(100) 79(35)	1041	1.04 (0.06)	1.00 (0.04)	1.04 (0.04)	1.04 (0.04)	0.22 (0.01)	0.18 (0.01)
14	benzeneethanol	1114	0.45 (0.03)	0.43 (0.03)	0.15 (0.01)	0.11 (0.01)	0.69 (0.03)	0.68 (0.04)
15	β-cyclocitral	1222	0.17 (0.02)	0.19 (0.01)	0.15 (0.01)	0.15 (0.01)	-	-
16	phenoxyethanol	1226	1.65 (0.06)	1.67 (0.05)	0.72 (0.03)	0.75 (0.04)	0.22 (0.01)	0.23 (0.01)
17	189[M+](3) 121(100) 93(90)	1322	0.43 (0.03)	0.42 (0.02)	0.16 (0.01)	0.14 (0.01)	0.08 (0.01)	0.09 (0.01)
18	bicycloelemene	1325	2.40 (0.07)	2.37 (0.05)	1.62 (0.06)	1.35 (0.06)	1.00 (0.05)	1.06 (0.05)
19	202[M+](10) 96(100) 81(85)	1350	4.50 (0.08)	4.49 (0.06)	5.85 (0.08)	5.12 (0.09)	0.34 (0.02)	0.32 (0.02)
20	anastreptene	1370	6.77 (0.10)	6.75 (0.07)	9.27 (0.11)	9.33 (0.11)	14.13 (0.12)	14.95 (0.13)
21	α-ylangene	1373	0.14 (0.01)	0.14 (0.01)	0.29 (0.02)	0.35 (0.03)	0.35 (0.02)	0.43 (0.03)
22	β-elemene	1391	0.58 (0.03)	0.59 (0.02)	0.55 (0.03)	0.54 (0.03)	0.73 (0.04)	0.74 (0.05)
23	α-gurjunene	1419	0.90 (0.04)	0.90 (0.03)	0.63 (0.04)	0.65 (0.04)	0.31 (0.02)	0.30 (0.02)
24	204[M+](25) 105(100) 91(92)	1423	0.78 (0.04)	0.73 (0.03)	0.39 (0.03)	0.40 (0.03)	0.39 (0.02)	0.39 (0.03)
25	(-)-aristolene	1427	0.49 (0.03)	0.43 (0.02)	0.29 (0.02)	0.31 (0.02)	0.16 (0.01)	0.12 (0.01)
26	γ-maaliene	1430	1.45 (0.06)	1.30 (0.05)	0.72 (0.04)	0.75 (0.04)	0.28 (0.01)	0.28 (0.02)
27	α-maaliene	1438	0.65 (0.04)	0.58 (0.03)	0.56 (0.03)	0.51 (0.04)	0.32 (0.02)	0.30 (0.02)
28	alloaromadendrene	1457	4.06 (0.08)	3.92 (0.06)	4.21 (0.07)	4.86 (0.08)	3.01 (0.07)	3.02 (0.09)
29	γ-gurjunene	1463	2.79 (0.06)	2.76 (0.05)	1.39 (0.05)	1.41 (0.05)	0.61 (0.04)	0.65 (0.05)
30	204[M+](13) 105(100) 91(85)	1469	0.19 (0.02)	0.20 (0.01)	0.55 (0.04)	0.65 (0.03)	0.11 (0.01)	0.11 (0.01)
31	204[M+](18) 91(100) 105(92)	1471	0.48 (0.03)	0.45 (0.02)	1.01 (0.05)	1.04 (0.05)	0.36 (0.02)	0.37 (0.02)
32	germacrene D	1474	0.43 (0.03)	0.42 (0.02)	0.56 (0.03)	0.55 (0.03)	0.29 (0.02)	0.33 (0.02)
33	ledene	1476	3.75 (0.06)	3.70 (0.06)	2.26 (0.07)	2.48 (0.06)	1.69 (0.05)	1.85 (0.05)
34	bicyclogermacrene	1488	25.78 (0.15)	25.44 (0.22)	33.60 (0.22)	33.23 (0.25)	21.48 (0.16)	20.92 (0.15)
35	γ-humulene	1493	0.83 (0.04)	0.80 (0.03)	2.07 (0.06)	2.16 (0.06)	1.68 (0.05)	1.73 (0.05)
36	cuparene	1502	1.51 (0.05)	1.57 (0.05)	4.11 (0.06)	4.30 (0.08)	1.81 (0.05)	1.87 (0.06)
37	α-bulnesene	1505	0.54 (0.04)	0.55 (0.03)	0.65 (0.04)	0.68 (0.04)	0.83 (0.03)	0.83 (0.04)
38	valencene	1510	0.53 (0.03)	0.53 (0.03)	-	-	0.19 (0.01)	0.23 (0.02)
39	4,5,9,10-dehydro-isolongifolene	1544	0.39 (0.03)	0.42 (0.02)	0.39 (0.03)	0.45 (0.04)	0.22 (0.01)	0.24 (0.02)
40	218[M+](5) 173(100) 145(97)	1555	0.82 (0.05)	0.80 (0.03)	0.46 (0.03)	0.48 (0.03)	0.14 (0.01)	0.14 (0.01)
41	spathulenol	1570	1.97 (0.06)	1.94 (0.06)	0.77 (0.04)	0.69 (0.05)	0.38 (0.02)	0.39 (0.03)
42	222[M+](5) 107(100) 147(75)	1573	1.32 (0.06)	1.22 (0.05)	0.70 (0.04)	0.70 (0.04)	0.30 (0.01)	0.33 (0.02)
43	220[M+](2) 159(100) 96(75)	1576	2.40 (0.07)	2.36 (0.07)	0.86 (0.04)	0.88 (0.05)	0.24 (0.02)	0.23 (0.01)
44	globulol	1590	1.51 (0.06)	1.57 (0.05)	0.62 (0.04)	0.62 (0.04)	-	-
45	220[M+](2) 120(100) 105(55)	1593	0.71 (0.04)	0.70 (0.03)	1.21 (0.06)	1.22 (0.06)	2.40 (0.05)	2.14 (0.09)
46	bisabola-2,10-diene 1,9-oxide	1602	1.53 (0.06)	1.53 (0.04)	0.63 (0.04)	0.61 (0.03)	0.75 (0.04)	0.81 (0.05)
47	220[M+](6) 119(100) 91(88)	1606	2.62 (0.07)	2.51 (0.05)	0.77 (0.05)	0.75 (0.04)	0.26 (0.01)	0.23 (0.02)
48	220[M+](2) 94(100) 109(85)	1613	1.76 (0.06)	1.73 (0.04)	1.25 (0.06)	1.28 (0.05)	0.91 (0.03)	0.96 (0.04)
49	ledene oxide-(II)	1629	2.71 (0.08)	2.73 (0.06)	4.42 (0.08)	4.47 (0.09)	0.90 (0.03)	0.86 (0.04)
50	isospathulenol	1631	2.59 (0.07)	2.70 (0.05)	-	-	1.20 (0.05)	1.05 (0.06)
51	α-acorenol	1633	0.52 (0.04)	0.57 (0.03)	0.33 (0.02)	0.36 (0.02)	0.31 (0.02)	0.31 (0.02)
52	234[M+](2) 105(100) 91(96)	1664	0.42 (0.03)	0.44 (0.02)	1.01 (0.04)	1.07 (0.05)	0.31 (0.01)	0.36 (0.03)
53	234[M+](3) 109(100) 91(85)	1672	0.75 (0.04)	0.87 (0.03)	0.71 (0.04)	0.75 (0.04)	0.48 (0.04)	0.46 (0.04)
54	aromadendrane-4,10-diol	1683	0.33 (0.03)	0.39 (0.02)	0.17 (0.01)	0.23 (0.01)	1.50 (0.05)	1.41 (0.05)
55	234[M+](4) 41(100) 109(98)	1686	0.71 (0.05)	0.78 (0.03)	0.24 (0.01)	0.25 (0.02)	0.40 (0.02)	0.39 (0.03)
56	232[M+](1) 105(100) 159(60)	1691	1.40 (0.06)	1.53 (0.05)	0.85 (0.04)	0.98 (0.05)	-	-
57	236[M+](1) 43(100) 139(65)	1694	1.19 (0.05)	1.23 (0.04)	2.80 (0.06)	2.95 (0.08)	29.24 (0.19)	29.05 (0.21)
58	1,4-dimethyl-7-isopropyl-azulene	1772	0.31 (0.03)	0.31 (0.01)	0.32 (0.02)	0.31 (0.02)	0.81 (0.04)	0.74 (0.04)
59	276[M+](1) 173(100) 109(50)	1805	2.28 (0.07)	2.29 (0.06)	0.11 (0.01)	0.12 (0.01)	-	-
60	276[M+] (8) 95(100) 43(66)	1818	0.31 (0.03)	0.35 (0.02)	0.21 (0.01)	0.23 (0.01)	-	-
61	280[M+](1) 110(100) 91(50)	1849	0.41 (0.03)	0.40 (0.02)	0.20 (0.02)	0.21 (0.02)	2.50 (0.05)	2.45 (0.06)
62	280[M+](1) 147(100) 119(70)	1924	0.29 (0.03)	0.32 (0.02)	0.13 (0.01)	0.11 (0.01)	0.09 (0.01)	0.10 (0.01)
63	geranyllinalool	2034	0.14 (0.01)	0.14 (0.01)	0.33 (0.02)	0.36 (0.02)	0.18 (0.01)	0.16 (0.01)
64	280[M+](2) 91(100) 105(85)	2041	0.29 (0.02)	0.28 (0.02)	-	-	0.11 (0.01)	0.14 (0.01)
65	280[M+](1) 91(100) 77(59)	2063	0.15 (0.02)	0.16 (0.01)	-	-	-	-
	Total		99.57(2.89)	99.08 (2.34)	98.42 (2.33)	99.05 (2.43)	99.07 (1.81)	99.05 (2.10)
	% Identified		74.34 (1.88)	73.84 (1.56)	77.94 (1.57)	78.70 (1.64)	60.17 (1.24)	60.61 (1.42)
	Including the following:							
	Aliphatics		1.55 (0.12)	1.52 (0.08)	-	-	0.77 (0.04)	0.74 (0.04)
	Aromatics		5.05 (0.22)	5.12 (0.18)	2.48 (012)	2.43 (0.12)	3.25 (0.11)	3.33 (0.14)
	Monoterpene hydrocarbons		2.14 (0.15)	2.17 (0.11)	4.71 (0.20)	4.73 (0.19)	1.01 (0.06)	0.95 (0.06)
	Sesquiterpene hydrocarbons		54.31 (0.99)	53.47 (0.88)	63.49 (1.00)	64.21 (1.07)	49.91 (0.81)	50.59 (0.91)
	Sesquiterpenoide hydrocarbons		11.28 (0.40)	11.56 (0.31)	7.26 (0.25)	7.34 (0.27)	5.22 (0.23)	4.99 (0.27)

- Less than 0.01%. ^a^ Retention index on Quadrex 007-5MS column. * The average value calculated based on the values from Table S1a–e and Table S2a–e for individual *C. sphagnicola* groups divided into 2021 and 2022. ( ) Standard deviation.

## Data Availability

The data presented in this study are available from the corresponding author upon request.

## Supplementary Materials

The following supporting information can be downloaded at: https://www.mdpi.com/article/10.3390/molecules30173642/s1, Figure S1: Microscopic images of the (a) leaves, (b) underleaves, and (c) cells with oil bodies of *C. sphagnicola* f. *sphagnicola* (1), *C. sphagnicola* f. *paludosa* (2), and *C. sphagnicola* LC (3). Table S1: (a) Volatile compounds detected in samples CSS-1–CSS-4, (b) volatile compounds detected in samples CSS-5–CSS-8, (c) volatile compounds detected in samples CSP-1–CSP-4, (d) volatile compounds detected in samples CSP-5–CSP-8, and (e) volatile compounds detected in samples CSL-1–CSL-3. Table S2: (a) Volatile compounds detected in samples CSS-9–CSS-12, (b) volatile compounds detected in samples CSS-13–CSS-16, (c) volatile compounds detected in samples CSP-9–CSP-12, (d) volatile compounds detected in samples CSP-13–CSP-16, and (e) volatile compounds detected in samples CSL-4–CSL-6. Table S3: Chemical Abstracts Service registry number (CAS) and methods used to identify compounds, including volatile compounds, detected in samples of *C. sphagnicola* f. *sphagnicola* (CSS), *C. sphagnicola* f. *paludosa* (CSP), and *C. sphagnicola* LC (CSL). Table S4: One-way ANOVA and post hoc Scheffé test showing statistically significant differences between genetic groups of *C. sphagnicola*: *C. sphagnicola* f. *sphagnicola* (CSS), *C. sphagnicola* f. *paludosa* (CSP), and *C. sphagnicola* LC (CSL). Table shows *p*-values. Figure S2: (a) Pie charts showing groups of chemical compounds detected in the *C. sphagnicola* f. *sphagnicola* (CSS) samples collected in 2021 and 2022, (b) pie charts showing groups of chemical compounds detected in the *C. sphagnicola* f. *palludosa* (CSP) samples collected in 2021 and 2022, and (c) pie charts showing groups of chemical compounds detected in the *C. sphagnicola* LC (CSL) samples collected in 2021 and 2022. Figure S3: (a) Linear plot of the factor lodgings for the first principal component PC1 based on all the detected VOCs in the *C. sphagnicola* samples; (b) linear plot of the lodgings for the second principal component PC2 based on all the detected VOCs in the *C. sphagnicola* samples. Figure S4: Heatmap clustering of VOC profiles from *C. sphagnicola* samples: *C. sphagnicola* f. *sphagnicola* (CSS), *C. sphagnicola* f. *paludosa* (CSP), and *C. sphagnicola* LC (CSL). The annotation bar shows the clustering of the samples by collection year (class). Each cell was colored based on the level of the concentration of the chemical compound in the sample. Figure S5: Dendrogram showing the results of the hierarchical cluster analysis of *C. sphagnicola* samples (*C. sphagnicola* f. *sphagnicola* (CSS), *C. sphagnicola* f. *paludosa* (CSP), and *C. sphagnicola* LC (CSL)) constructed based on the Euclidean distance and Ward’s linkage method using all the detected VOCs in the samples collected in 2021 and 2022. Figure S6: Variable importance in projection (VIP) identified by PLS-DA. The red and blue boxes on the right indicate whether the compound concentration was increased (red) or decreased (blue) in the samples of the three studied groups of *C. sphagnicola*: *C. sphagnicola* f. *sphagnicola* (CSS), *C. sphagnicola* f. *paludosa* (CSP), and *C. sphagnicola* LC (CSL). Table S5: The *C. sphagnicola* f. *sphagnicola* sampling data in the 2021 year used for the study. Table S6: The *C. sphagnicola* f. *paludosa* sampling data in the 2021 year used for the study. Table S7: The *C. sphagnicola* LC sampling data in the 2021 year used for the study. Table S8: The *C. sphagnicola* f. *sphagnicola* sampling data in the 2022 year used for the study. Table S9: The *C. sphagnicola* f. *paludosa* sampling data in the 2022 year used for the study. Table S10: The *C. sphagnicola* LC sampling data in the 2022 year used for the study.
