# Supplementary material for: The Composition of Volatile Organic Compounds Correlates with the Genetic Variability Within the Calypogeia sphagnicola Species Complex (Marchantiophyta, Calypogeiaceae)
Source: Molecules. 2025 Sep 7;30(17):3642. doi: 10.3390/molecules30173642 (PMC12430087; doi:10.3390/molecules30173642)
Supplement: Supplementary file 1 [file molecules-30-03642-s001.zip › molecules-3783057-supplementary.pdf]

molecules

MDPI

Supplementary Material

The composition of volatile organic compounds correlates with the genetic variability within the *Calypogeia sphagnicola* species complex (Marchantiophyta, Calypogeiaceae)

Rafał Wawrzyniak [1,*], Małgorzata Guzowska [1], Katarzyna Buczkowska [2,*] and Alina Bączkiewicz [2]

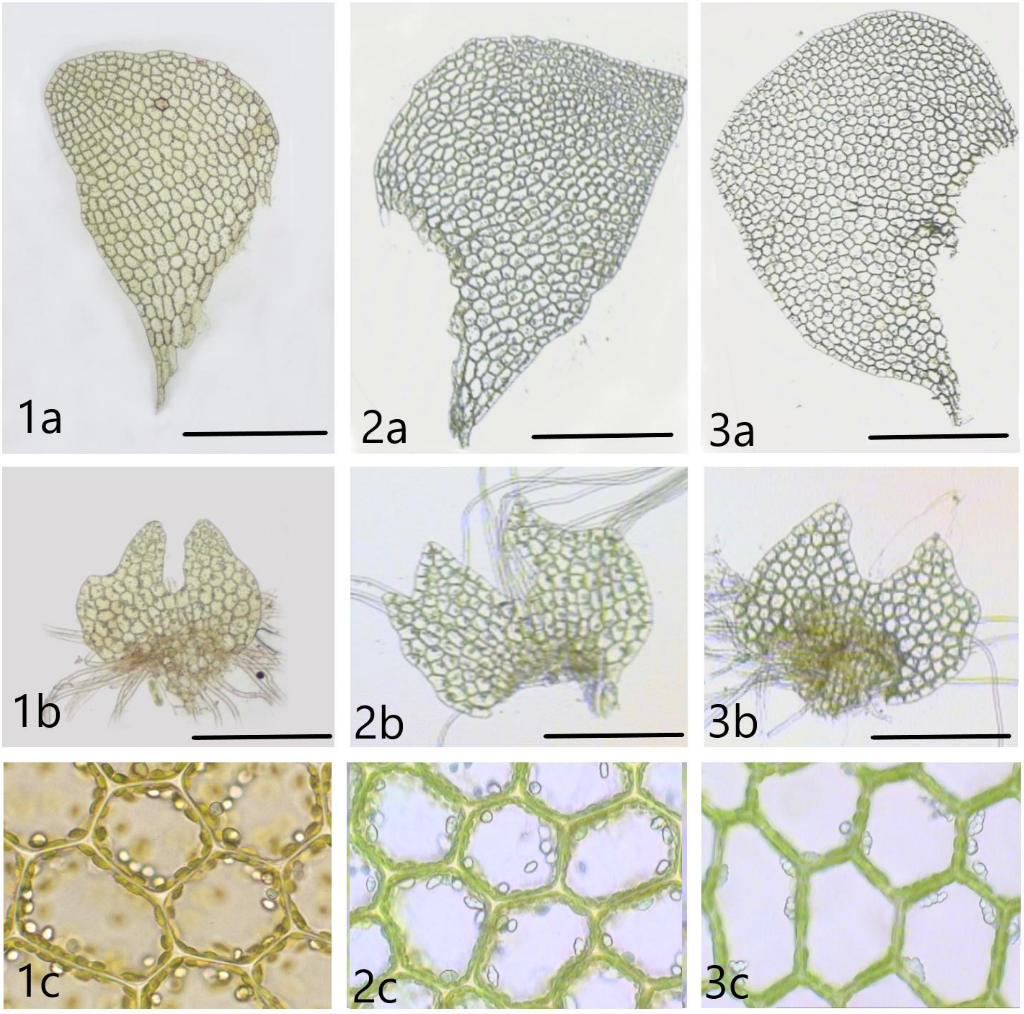

Figure S1. Microscopic images of the a) leaves, b) under leaves and c) cells with oil bodies of *C. sphagnicola* f. *sphagnicola* (1), *C. sphagnicola* f. *paludosa* (2), *C. sphagnicola* LC (3)

Table S1. (a) Volatile compounds detected in samples CSS-1 – CSS-4.

No.	Compounds	RI [a]	Code [b]			
			CSS-1	CSS-2	CSS-3	CSS-4
1	3-hydroxybutan-2-one	<700	0.40 (0.02)	0.40 (0.03)	0.30 (0.02)	0.35 (0.02)
2	3-methylbutan-1-ol	706	0.20 (0.03)	0.30 (0.02)	0.30 (0.02)	0.25 (0.02)
3	hexan-1-ol	867	0.20 (0.02)	0.30 (0.02)	0.30 (0.03)	0.30 (0.03)
4	tricyclene	926	0.06 (0.01)	0.04 (0.01)	0.06 (0.01)	0.05 (0.01)
5	α-pinene	936	0.40 (0.03)	0.40 (0.03)	0.39 (0.04)	0.28 (0.03)
6	2-methylpentan-2,4-diol	938	0.70 (0.03)	0.30 (0.02)	0.50 (0.03)	0.30 (0.02)
7	camphene	953	0.40 (0.02)	0.60 (0.05)	0.80 (0.05)	0.57 (0.04)
8	benzaldehyde	960	0.80 (0.03)	0.90 (0.04)	1.15 (0.06)	0.60 (0.04)
9	β-pinene	978	0.98 (0.04)	0.90 (0.05)	1.00 (0.06)	0.80 (0.05)
10	7-octen-4-ol	982	0.40 (0.02)	0.30 (0.03)	0.30 (0.02)	0.30 (0.02)
11	3-carene	1009	-	-	-	-
12	benzenemethanol	1023	1.40 (0.09)	1.90 (0.07)	1.87 (0.08)	1.99 (0.07)
13	120[M+](16) 91(100) 79(35)	1041	1.20 (0.07)	1.10 (0.05)	0.87 (0.06)	0.86 (0.05)
14	benzeneethanol	1114	0.60 (0.04)	0.40 (0.03)	0.30 (0.03)	0.40 (0.03)
15	β-cyclocitral	1222	0.10 (0.02)	0.10 (0.01)	0.18 (0.02)	0.16 (0.02)
16	phenoxyethanol	1226	1.60 (0.06)	1.60 (0.04)	1.80 (0.07)	1.40 (0.07)
17	189[M+](3) 121(100) 93(90)	1322	0.60 (0.03)	0.55 (0.03)	0.30 (0.03)	0.30 (0.03)
18	bicycloelemene	1325	2.70 (0.08)	2.50 (0.07)	2.10 (0.09)	2.30 (0.08)
19	202[M+](10) 96(100) 81(85)	1350	4.60 (0.06)	4.10 (0.08)	4.50 (0.08)	4.70 (0.09)
20	anastreptene	1370	7.10 (0.09)	7.50 (0.10)	7.03 (0.11)	7.12 (0.10)
21	α-ylangene	1373	0.10 (0.01)	0.10 (0.01)	0.15 (0.02)	0.10 (0.01)
22	β-elemene	1391	0.55 (0.02)	0.60 (0.03)	0.58 (0.04)	0.56 (0.03)
23	α-gurjunene	1419	0.90 (0.04)	0.90 (0.04)	0.80 (0.06)	0.98 (0.04)
24	204[M+](25) 105(100) 91(92)	1423	0.60 (0.03)	0.70 (0.03)	0.90 (0.05)	0.80 (0.04)
25	(-)-aristolene	1427	0.40 (0.02)	0.30 (0.02)	0.50 (0.04)	0.80 (0.05)
26	γ-maaliene	1430	1.70 (0.04)	1.70 (0.05)	1.30 (0.06)	1.20 (0.06)
27	α-maaliene	1438	0.64 (0.03)	0.70 (0.06)	0.60 (0.04)	0.78 (0.04)
28	alloaromadendrene	1457	3.31 (0.06)	4.10 (0.08)	4.50 (0.08)	4.83 (0.08)
29	γ-gurjunene	1463	2.59 (0.05)	3.00 (0.06)	2.78 (0.06)	2.80 (0.06)
30	204[M+](13) 105(100) 91(85)	1469	0.20 (0.02)	0.10 (0.01)	0.15 (0.02)	0.23 (0.03)
31	204[M+](18) 91(100) 105(92)	1471	0.50 (0.03)	0.40 (0.02)	0.42 (0.03)	0.39 (0.03)
32	germacrene D	1474	0.50 (0.02)	0.30 (0.02)	0.50 (0.04)	0.40 (0.03)
33	ledene	1476	3.70 (0.04)	3.90 (0.06)	4.03 (0.07)	4.23 (0.06)
34	bicyclogermacrene	1488	26.80 (0.13)	25.70 (0.10)	25.50 (0.14)	25.70 (0.13)
35	γ-humulene	1493	0.60 (0.04)	0.70 (0.05)	0.79 (0.05)	0.87 (0.04)
36	cuparene	1502	1.60 (0.03)	1.45 (0.07)	1.50 (0.06)	1.80 (0.06)
37	α-bulnesene	1505	0.53 (0.02)	0.49 (0.03)	0.57 (0.04)	0.52 (0.03)
38	valencene	1510	0.61 (0.04)	0.60 (0.04)	0.55 (0.04)	0.49 (0.03)
39	4,5,9,10-dehydro-isolongifolene	1544	0.33 (0.03)	0.30 (0.02)	0.36 (0.03)	0.38 (0.02)
40	218[M+](5) 173(100) 145(97)	1555	0.80 (0.05)	0.65 (0.05)	0.73 (0.06)	0.92 (0.04)
41	spathulenol	1570	2.10 (0.04)	1.90 (0.06)	1.93 (0.07)	1.86 (0.05)
42	222[M+](5) 107(100) 147(75)	1573	1.20 (0.06)	1.00 (0.05)	1.10 (0.06)	1.50 (0.04)
43	220[M+](2) 159(100) 96(75)	1576	2.50 (0.05)	2.20 (0.07)	2.30 (0.08)	2.18 (0.06)
44	globulol	1590	1.10 (0.04)	1.60 (0.05)	1.10 (0.06)	1.50 (0.04)
45	220[M+](2) 120(100) 105(55)	1593	0.53 (0.03)	0.80 (0.04)	0.60 (0.04)	0.59 (0.03)
46	bisabola-2,10-diene 1,9-oxide	1602	1.40 (0.06)	1.20 (0.05)	1.10 (0.05)	1.80 (0.06)
47	220[M+](6) 119(100) 91(88)	1606	2.20 (0.07)	2.90 (0.06)	2.50 (0.06)	2.20 (0.07)
48	220[M+](2) 94(100) 109(85)	1613	1.70 (0.05)	1.48 (0.05)	2.00 (0.07)	1.68 (0.06)
49	ledene oxide-(II)	1629	3.30 (0.08)	2.70 (0.07)	2.53 (0.08)	2.90 (0.09)
50	isospathulenol	1631	2.70 (0.06)	3.00 (0.06)	2.43 (0.07)	2.56 (0.08)
51	α-acorenol	1633	0.57 (0.03)	0.60 (0.04)	0.52 (0.04)	0.50 (0.03)
52	234[M+](2) 105(100) 91(96)	1664	0.32 (0.02)	0.30 (0.02)	0.42 (0.03)	0.36 (0.02)
53	234[M+](3) 109(100) 91(85)	1672	0.70 (0.03)	0.90 (0.03)	0.84 (0.06)	0.70 (0.04)
54	aromadendrane-4,10-diol	1683	0.23 (0.02)	0.30 (0.02)	0.36 (0.03)	0.38 (0.03)

55	234[M+](4) 41(100) 109(98)	1686	0.68 (0.04)	0.70 (0.04)	0.91 (0.05)	0.84 (0.05)
56	232[M+](1) 105(100) 159(60)	1691	1.34 (0.05)	1.22 (0.06)	1.56 (0.08)	1.10 (0.07)
57	236[M+](1) 43(100) 139(65)	1694	1.20 (0.04)	1.10 (0.05)	1.40 (0.06)	1.30 (0.06)
58	1,4-dimethyl-7-isopropyl-azulene	1772	0.40 (0.02)	0.32 (0.03)	0.32 (0.03)	0.27 (0.02)
59	276[M+](1) 173(100) 109(50)	1805	2.90 (0.07)	2.40 (0.06)	2.60 (0.07)	2.31 (0.09)
60	276[M+] (8) 95(100) 43(66)	1818	0.30 (0.03)	0.48 (0.03)	0.23 (0.04)	0.29 (0.03)
61	280[M+](1) 110(100) 91(50)	1849	0.25 (0.02)	0.39 (0.03)	0.36 (0.03)	0.32 (0.03)
62	280[M+](1) 147(100) 119(70)	1924	0.23 (0.02)	0.32 (0.03)	0.30 (0.03)	0.30 (0.02)
63	geranyllinalool	2034	0.10 (0.01)	0.30 (0.02)	0.10 (0.01)	0.10 (0.01)
64	280[M+](2) 91(100) 105(85)	2041	0.20 (0.02)	0.54 (0.04)	0.30 (0.02)	0.37 (0.04)
65	280[M+](1) 91(100) 77(59)	2063	0.20 (0.03)	0.30 (0.03)	0.10 (0.01)	0.10 (0.01)
	Total		99.75 (2.53)	99.83 (2.72)	99.17 (3.17)	99.82 (2.86)
	% Identified		74.80 (1.61)	75.20 (1.76)	73.78 (2.05)	75.48 (1.83)
	Including the following:					
	Aliphatics		1.90 (0.12)	1.60 (0.12)	1.70 (0.12)	1.50 (0.11)
	Aromatics		4.40 (0.22)	4.80 (0.18)	5.12 (0.24)	4.39 (0.21)
	Monoterpene hydrocarbons		1.94 (0.12)	2.04 (0.15)	2.43 (0.18)	1.86 (0.15)
	Sesquiterpene hydrocarbons		55.06 (0.81)	55.16 (0.94)	54.46 (1.10)	56.13 (0.97)
	Sesquiterpenoide hydrocarbons		11.50 (0.34)	11.60 (0.37)	10.07 (0.41)	11.60 (0.39)

- less than 0.01%. [a] Retention index on Quadrex 007-5MS column. [b] For abbreviations of samples see Table S5. Mean % and ( ) standard deviation for sample analyzed in triplicate.

Table S1. (b) Volatile compounds detected in samples CSS-5 – CSS-8.

No.	Compounds	RI [a]	Code [b]			
			CSS-5	CSS-6	CSS-7	CSS-8
1	3-hydroxybutan-2-one	<700	0.25 (0.02)	0.20 (0.02)	0.35 (0.02)	0.30 (0.03)
2	3-methylbutan-1-ol	706	0.35 (0.02)	0.15 (0.01)	0.20 (0.02)	0.25 (0.03)
3	hexan-1-ol	867	0.25 (0.03)	0.27 (0.03)	0.30 (0.03)	0.30 (0.02)
4	tricyclene	926	0.07 (0.01)	0.04 (0.01)	0.03 (0.01)	0.05 (0.01)
5	α-pinene	936	0.30 (0.03)	0.41 (0.03)	0.46 (0.03)	0.45 (0.04)
6	2-methylpentan-2,4-diol	938	0.30 (0.02)	0.30 (0.02)	0.30 (0.03)	0.55 (0.04)
7	camphene	953	0.49 (0.04)	0.41 (0.03)	0.61 (0.04)	0.61 (0.03)
8	benzaldehyde	960	1.20 (0.05)	1.22 (0.07)	1.08 (0.05)	1.13 (0.08)
9	β-pinene	978	0.90 (0.05)	0.95 (0.05)	1.18 (0.07)	1.07 (0.06)
10	7-octen-4-ol	982	0.20 (0.02)	0.30 (0.02)	0.30 (0.02)	0.30 (0.02)
11	3-carene	1009	-	-	-	-
12	benzenemethanol	1023	2.04 (0.08)	3.20 (0.09)	1.65 (0.06)	1.53 (0.09)
13	120[M+](16) 91(100) 79(35)	1041	0.97 (0.04)	1.20 (0.05)	1.02 (0.04)	1.09 (0.08)
14	benzeneethanol	1114	0.27 (0.03)	0.68 (0.04)	0.46 (0.04)	0.45 (0.03)
15	β-cyclocitral	1222	0.14 (0.02)	0.41 (0.03)	0.16 (0.01)	0.12 (0.01)
16	phenoxyethanol	1226	2.10 (0.05)	1.22 (0.04)	1.51 (0.04)	1.98 (0.07)
17	189[M+](3) 121(100) 93(90)	1322	0.40 (0.03)	0.39 (0.03)	0.47 (0.03)	0.43 (0.03)
18	bicycloelemene	1325	3.20 (0.06)	1.89 (0.05)	2.23 (0.05)	2.31 (0.11)
19	202[M+](10) 96(100) 81(85)	1350	4.40 (0.07)	4.60 (0.08)	4.38 (0.08)	4.75 (0.10)
20	anastreptene	1370	6.63 (0.09)	5.72 (0.09)	6.83 (0.09)	6.25 (0.13)
21	α-ylangene	1373	0.12 (0.01)	0.27 (0.03)	0.15 (0.01)	0.13 (0.01)
22	β-elemene	1391	0.62 (0.04)	0.54 (0.03)	0.56 (0.03)	0.59 (0.04)
23	α-gurjunene	1419	1.05 (0.05)	0.94 (0.05)	0.87 (0.04)	0.76 (0.03)
24	204[M+](25) 105(100) 91(92)	1423	1.20 (0.04)	0.81 (0.06)	0.61 (0.05)	0.61 (0.04)
25	(-)-aristolene	1427	0.60 (0.03)	0.40 (0.03)	0.46 (0.04)	0.45 (0.03)
26	γ-maaliene	1430	1.90 (0.05)	1.08 (0.06)	1.38 (0.06)	1.37 (0.09)
27	α-maaliene	1438	0.53 (0.03)	0.67 (0.04)	0.63 (0.04)	0.61 (0.04)
28	alloaromadendrene	1457	4.50 (0.07)	3.60 (0.08)	3.84 (0.07)	3.81 (0.10)
29	γ-gurjunene	1463	2.93 (0.06)	2.95 (0.07)	2.92 (0.06)	2.38 (0.08)
30	204[M+](13) 105(100) 91(85)	1469	0.18 (0.01)	0.31 (0.02)	0.16 (0.01)	0.18 (0.02)
31	204[M+](18) 91(100) 105(92)	1471	0.49 (0.02)	0.67 (0.04)	0.47 (0.03)	0.46 (0.03)
32	germacrene D	1474	0.40 (0.03)	0.40 (0.03)	0.47 (0.03)	0.45 (0.03)
33	ledene	1476	4.10 (0.05)	3.12 (0.05)	3.46 (0.06)	3.44 (0.07)
34	bicyclogermacrene	1488	24.80 (0.16)	24.38 (0.14)	27.64 (0.18)	25.73 (0.21)
35	γ-humulene	1493	0.93 (0.04)	0.94 (0.04)	0.92 (0.04)	0.91 (0.04)
36	cuparene	1502	1.60 (0.05)	1.35 (0.05)	1.38 (0.06)	1.37 (0.05)
37	α-bulnesene	1505	0.47 (0.04)	0.54 (0.05)	0.61 (0.04)	0.61 (0.04)
38	valencene	1510	0.53 (0.03)	0.27 (0.03)	0.63 (0.03)	0.57 (0.03)
39	4,5,9,10-dehydro-isolongifolene	1544	0.33 (0.03)	0.81 (0.04)	0.35 (0.02)	0.28 (0.03)
40	218[M+](5) 173(100) 145(97)	1555	0.90 (0.04)	1.08 (0.06)	0.71 (0.04)	0.73 (0.05)
41	spathulenol	1570	1.97 (0.06)	2.01 (0.05)	1.87 (0.06)	2.09 (0.09)
42	222[M+](5) 107(100) 147(75)	1573	1.50 (0.05)	1.49 (0.05)	1.38 (0.05)	1.37 (0.08)
43	220[M+](2) 159(100) 96(75)	1576	2.22 (0.07)	2.98 (0.07)	2.15 (0.08)	2.66 (0.11)
44	globulol	1590	1.80 (0.06)	1.49 (0.05)	1.69 (0.07)	1.80 (0.09)
45	220[M+](2) 120(100) 105(55)	1593	0.63 (0.04)	0.94 (0.04)	0.82 (0.04)	0.73 (0.05)
46	bisabola-2,10-diene 1,9-oxide	1602	1.90 (0.07)	1.49 (0.05)	1.68 (0.05)	1.67 (0.06)
47	220[M+](6) 119(100) 91(88)	1606	2.20 (0.08)	3.25 (0.06)	2.38 (0.07)	3.35 (0.06)
48	220[M+](2) 94(100) 109(85)	1613	1.40 (0.06)	2.17 (0.05)	1.84 (0.06)	1.83 (0.04)
49	ledene oxide-(II)	1629	2.31 (0.09)	2.74 (0.06)	2.54 (0.09)	2.62 (0.06)
50	isospathulenol	1631	2.60 (0.10)	2.21 (0.07)	2.79 (0.10)	2.41 (0.05)
51	α-acorenol	1633	0.40 (0.04)	0.60 (0.04)	0.49 (0.04)	0.45 (0.03)
52	234[M+](2) 105(100) 91(96)	1664	0.29 (0.03)	0.81 (0.05)	0.37 (0.04)	0.46 (0.03)
53	234[M+](3) 109(100) 91(85)	1672	0.60 (0.05)	0.70 (0.04)	0.76 (0.05)	0.76 (0.04)
54	aromadendrane-4,10-diol	1683	0.31 (0.02)	0.40 (0.03)	0.31 (0.03)	0.33 (0.03)

55	234[M+](4) 41(100) 109(98)	1686	0.65 (0.04)	0.67 (0.05)	0.61 (0.04)	0.61 (0.05)
56	232[M+](1) 105(100) 159(60)	1691	1.50 (0.05)	1.76 (0.06)	1.38 (0.06)	1.37 (0.06)
57	236[M+](1) 43(100) 139(65)	1694	1.00 (0.04)	1.30 (0.07)	1.10 (0.05)	1.10 (0.05)
58	1,4-dimethyl-7-isopropyl-azulene	1772	0.30 (0.03)	0.27 (0.03)	0.30 (0.02)	0.33 (0.02)
59	276[M+](1) 173(100) 109(50)	1805	2.10 (0.05)	1.80 (0.05)	2.12 (0.07)	2.00 (0.10)
60	276[M+] (8) 95(100) 43(66)	1818	0.30 (0.02)	0.31 (0.02)	0.27 (0.02)	0.31 (0.03)
61	280[M+](1) 110(100) 91(50)	1849	0.27 (0.02)	0.41 (0.02)	0.43 (0.03)	0.82 (0.05)
62	280[M+](1) 147(100) 119(70)	1924	0.30 (0.03)	0.40 (0.03)	0.15 (0.01)	0.34 (0.03)
63	geranyllinalool	2034	0.10 (0.01)	0.13 (0.01)	0.13 (0.01)	0.15 (0.01)
64	280[M+](2) 91(100) 105(85)	2041	0.36 (0.02)	0.23 (0.02)	0.15 (0.01)	0.15 (0.01)
65	280[M+](1) 91(100) 77(59)	2063	0.10 (0.01)	0.13 (0.01)	0.15 (0.01)	0.15 (0.01)
	Total		99.75 (2.78)	99.38 (2.87)	99.60 (2.86)	99.22 (3.31)
	% Identified		75.79 (1.87)	70.97 (1.84)	75.72 (1.89)	72.96 (2.16)
	Including the following:					
	Aliphatics		1.35 (0.11)	1.22 (0.10)	1.45 (0.12)	1.70 (0.14)
	Aromatics		5.61 (0.21)	6.32 (0.24)	4.70 (0.19)	5.09 (0.27)
	Monoterpene hydrocarbons		1.90 (0.15)	2.22 (0.15)	2.44 (0.16)	2.30 (0.15)
	Sesquiterpene hydrocarbons		55.54 (0.95)	50.14 (0.99)	55.63 (0.97)	52.35 (1.18)
	Sesquiterpenoide hydrocarbons		11.39 (0.45)	11.07 (0.36)	11.50 (0.45)	11.52 (0.42)

- less than 0.01%. [a] Retention index on Quadrex 007-5MS column. [b] For abbreviations of samples see Table S5. Mean % and ( ) standard deviation for sample analyzed in triplicate.

Table S1. (c) Volatile compounds detected in samples CSP-1 – CSP-4.

No.	Compounds	RI [a]	Code [b]			
			CSP-1	CSP-2	CSP-3	CSP-4
1	3-hydroxybutan-2-one	<700	-	-	-	-
2	3-methylbutan-1-ol	706	-	-	-	-
3	hexan-1-ol	867	-	-	-	-
4	tricyclene	926	0.33 (0.03)	0.40 (0.03)	0.26 (0.01)	0.23 (0.01)
5	α-pinene	936	1.78 (0.06)	1.51 (0.05)	1.50 (0.05)	1.40 (0.06)
6	2-methylpentan-2,4-diol	938	-	-	-	-
7	camphene	953	0.87 (0.04)	0.70 (0.03)	0.83 (0.04)	0.82 (0.04)
8	benzaldehyde	960	0.21 (0.02)	0.20 (0.01)	0.23 (0.01)	0.24 (0.01)
9	β-pinene	978	1.52 (0.06)	1.61 (0.05)	1.67 (0.06)	1.54 (0.06)
10	7-octen-4-ol	982	-	-	-	-
11	3-carene	1009	0.21 (0.02)	0.20 (0.02)	0.26 (0.01)	0.26 (0.01)
12	benzenemethanol	1023	1.30 (0.05)	1.01 (0.04)	1.30 (0.07)	1.17 (0.05)
13	120[M+](16) 91(100) 79(35)	1041	0.90 (0.04)	1.10 (0.04)	1.07 (0.03)	1.03 (0.05)
14	benzeneethanol	1114	0.10 (0.01)	0.10 (0.01)	0.12 (0.01)	0.16 (0.01)
15	β-cyclocitral	1222	0.10 (0.01)	0.10 (0.01)	0.19 (0.01)	0.15 (0.01)
16	phenoxyethanol	1226	0.76 (0.04)	0.60 (0.03)	0.59 (0.02)	0.82 (0.03)
17	189[M+](3) 121(100) 93(90)	1322	0.10 (0.01)	0.10 (0.01)	0.13 (0.01)	0.26 (0.01)
18	bicycloelemene	1325	2.08 (0.06)	1.51 (0.06)	1.18 (0.05)	1.31 (0.05)
19	202[M+](10) 96(100) 81(85)	1350	6.51 (0.09)	6.94 (0.09)	6.47 (0.07)	5.11 (0.07)
20	anastreptene	1370	8.51 (0.10)	8.80 (0.12)	9.59 (0.13)	9.82 (0.13)
21	α-ylangene	1373	0.28 (0.02)	0.30 (0.03)	0.26 (0.01)	0.29 (0.01)
22	β-elemene	1391	0.68 (0.04)	0.49 (0.03)	0.52 (0.02)	0.52 (0.02)
23	α-gurjunene	1419	0.43 (0.03)	0.59 (0.04)	0.78 (0.04)	0.67 (0.02)
24	204[M+](25) 105(100) 91(92)	1423	0.32 (0.03)	0.28 (0.02)	0.39 (0.03)	0.52 (0.02)
25	(-)-aristolene	1427	0.32 (0.02)	0.20 (0.01)	0.26 (0.01)	0.26 (0.02)
26	γ-maaliene	1430	0.76 (0.05)	0.64 (0.04)	0.52 (0.05)	0.65 (0.03)
27	α-maaliene	1438	0.65 (0.04)	0.64 (0.03)	0.39 (0.03)	0.39 (0.03)
28	alloaromadendrene	1457	4.40 (0.07)	3.70 (0.06)	4.43 (0.08)	4.93 (0.06)
29	γ-gurjunene	1463	1.52 (0.05)	1.41 (0.04)	1.70 (0.05)	1.40 (0.05)
30	204[M+](13) 105(100) 91(85)	1469	0.43 (0.03)	0.44 (0.03)	0.39 (0.03)	0.52 (0.03)
31	204[M+](18) 91(100) 105(92)	1471	0.97 (0.04)	0.90 (0.04)	0.78 (0.04)	1.04 (0.04)
32	germacrene D	1474	0.65 (0.04)	0.50 (0.03)	0.52 (0.03)	0.52 (0.04)
33	ledene	1476	1.83 (0.06)	1.80 (0.06)	2.36 (0.06)	2.35 (0.08)
34	bicyclogermacrene	1488	32.37 (0.24)	35.72 (0.21)	32.83 (0.19)	31.18 (0.27)
35	γ-humulene	1493	2.34 (0.05)	2.05 (0.05)	2.51 (0.06)	1.79 (0.05)
36	cuparene	1502	4.88 (0.06)	3.28 (0.07)	4.71 (0.04)	4.51 (0.07)
37	α-bulnesene	1505	0.63 (0.04)	0.70 (0.03)	0.65 (0.03)	0.65 (0.03)
38	valencene	1510	-	-	-	-
39	4,5,9,10-dehydro-isolongifolene	1544	0.32 (0.03)	0.40 (0.04)	0.52 (0.02)	0.36 (0.03)
40	218[M+](5) 173(100) 145(97)	1555	0.32 (0.03)	0.50 (0.03)	0.32 (0.02)	0.41 (0.03)
41	spathulenol	1570	0.97 (0.05)	0.91 (0.04)	0.80 (0.03)	0.57 (0.02)
42	222[M+](5) 107(100) 147(75)	1573	0.65 (0.04)	0.70 (0.03)	0.78 (0.02)	0.78 (0.04)
43	220[M+](2) 159(100) 96(75)	1576	0.86 (0.05)	0.73 (0.05)	0.91 (0.03)	0.97 (0.05)
44	globulol	1590	0.54 (0.04)	0.70 (0.03)	0.52 (0.04)	0.52 (0.04)
45	220[M+](2) 120(100) 105(55)	1593	1.41 (0.06)	1.21 (0.05)	1.24 (0.05)	1.17 (0.06)
46	bisabola-2,10-diene 1,9-oxide	1602	0.60 (0.04)	0.50 (0.03)	0.64 (0.04)	0.57 (0.04)
47	220[M+](6) 119(100) 91(88)	1606	0.80 (0.05)	0.70 (0.03)	0.78 (0.05)	0.84 (0.04)
48	220[M+](2) 94(100) 109(85)	1613	1.30 (0.06)	1.11 (0.04)	1.27 (0.07)	1.23 (0.06)
49	ledene oxide-(II)	1629	4.23 (0.08)	4.35 (0.08)	4.36 (0.10)	4.42 (0.09)
50	isospathulenol	1631	-	-	-	-
51	α-acorenol	1633	0.32 (0.03)	0.40 (0.02)	0.26 (0.02)	0.39 (0.02)
52	234[M+](2) 105(100) 91(96)	1664	0.97 (0.04)	0.90 (0.04)	1.05 (0.04)	1.04 (0.05)
53	234[M+](3) 109(100) 91(85)	1672	0.65 (0.04)	0.60 (0.03)	0.65 (0.03)	0.76 (0.04)
54	aromadendrane-4,10-diol	1683	0.10 (0.01)	0.10 (0.01)	0.26 (0.01)	0.19 (0.01)

55	234[M+](4) 41(100) 109(98)	1686	0.21 (0.02)	0.20 (0.01)	0.31 (0.01)	0.30 (0.02)
56	232[M+](1) 105(100) 159(60)	1691	0.65 (0.04)	0.90 (0.06)	0.97 (0.05)	0.76 (0.04)
57	236[M+](1) 43(100) 139(65)	1694	3.04 (0.08)	2.96 (0.07)	2.58 (0.07)	2.97 (0.06)
58	1,4-dimethyl-7-isopropyl-azulene	1772	0.54 (0.04)	0.40 (0.02)	0.26 (0.01)	0.26 (0.02)
59	276[M+](1) 173(100) 109(50)	1805	0.08 (0.01)	0.10 (0.01)	0.11 (0.01)	0.09 (0.01)
60	276[M+] (8) 95(100) 43(66)	1818	0.25 (0.02)	0.20 (0.01)	0.18 (0.01)	0.22 (0.01)
61	280[M+](1) 110(100) 91(50)	1849	0.21 (0.02)	0.10 (0.01)	0.23 (0.01)	0.19 (0.02)
62	280[M+](1) 147(100) 119(70)	1924	0.10 (0.01)	0.09 (0.01)	0.19 (0.02)	0.17 (0.01)
63	geranyllinalool	2034	0.44 (0.03)	0.30 (0.01)	0.33 (0.03)	0.32 (0.02)
64	280[M+](2) 91(100) 105(85)	2041	-	-	-	-
65	280[M+](1) 91(100) 77(59)	2063	-	-	-	-
	Total		98.30 (2.47)	97.58 (2.18)	98.91 (2.17)	96.01 (2.30)
	% Identified		77.57 (1.66)	76.82 (1.47)	78.11 (1.47)	75.63 (1.54)
	Including the following:					
	Aliphatics		-	-	-	-
	Aromatics		2.37 (0.12)	1.91 (0.09)	2.24 (0.11)	2.39 (0.10)
	Monoterpene hydrocarbons		4.81 (0.22)	4.52 (0.19)	4.71 (0.18)	4.40 (0.19)
	Sesquiterpene hydrocarbons		63.19 (1.04)	63.13 (0.97)	63.99 (0.91)	61.86 (1.01)
	Sesquiterpenoide hydrocarbons		7.20 (0.28)	7.26 (0.22)	7.17 (0.27)	6.98 (0.24)

- less than 0.01%. [a] Retention index on Quadrex 007-5MS column. [b] For abbreviations of samples see Table S6. Mean % and ( ) standard deviation for sample analyzed in triplicate.

Table S1. (d) Volatile compounds detected in samples CSP-5 – CSP-8.

No.	Compounds	RI [a]	Code [b]			
			CSP-5	CSP-6	CSP-7	CSP-8
1	3-hydroxybutan-2-one	<700	-	-	-	-
2	3-methylbutan-1-ol	706	-	-	-	-
3	hexan-1-ol	867	-	-	-	-
4	tricyclene	926	0.25 (0.03)	0.51 (0.04)	0.49 (0.03)	0.25 (0.02)
5	α-pinene	936	1.50 (0.05)	1.63 (0.06)	1.64 (0.05)	1.50 (0.04)
6	2-methylpentan-2,4-diol	938	-	-	-	-
7	camphene	953	0.90 (0.04)	0.89 (0.05)	0.99 (0.04)	0.79 (0.03)
8	benzaldehyde	960	0.30 (0.02)	0.31 (0.02)	0.53 (0.04)	0.47 (0.03)
9	β-pinene	978	1.89 (0.06)	1.50 (0.07)	1.60 (0.06)	1.28 (0.05)
10	7-octen-4-ol	982	-	-	-	-
11	3-carene	1009	0.32 (0.03)	0.21 (0.01)	0.21 (0.01)	0.21 (0.01)
12	benzenemethanol	1023	1.14 (0.05)	1.50 (0.03)	1.72 (0.04)	1.23 (0.05)
13	120[M+](16) 91(100) 79(35)	1041	1.14 (0.05)	0.97 (0.04)	0.99 (0.04)	1.09 (0.04)
14	benzeneethanol	1114	0.25 (0.02)	0.14 (0.01)	0.11 (0.01)	0.22 (0.01)
15	β-cyclocitral	1222	0.11 (0.01)	0.21 (0.01)	0.12 (0.01)	0.22 (0.01)
16	phenoxyethanol	1226	0.78 (0.04)	0.80 (0.03)	0.83 (0.04)	0.58 (0.03)
17	189[M+](3) 121(100) 93(90)	1322	0.12 (0.01)	0.19 (0.01)	0.17 (0.01)	0.19 (0.01)
18	bicycloelemene	1325	1.27 (0.05)	1.95 (0.06)	1.85 (0.05)	1.84 (0.06)
19	202[M+](10) 96(100) 81(85)	1350	5.11 (0.09)	5.06 (0.08)	5.31 (0.09)	6.31 (0.07)
20	anastreptene	1370	8.83 (0.11)	9.94 (0.10)	8.90 (0.11)	9.79 (0.09)
21	α-ylangene	1373	0.25 (0.02)	0.40 (0.03)	0.23 (0.02)	0.33 (0.02)
22	β-elemene	1391	0.50 (0.03)	0.51 (0.03)	0.67 (0.04)	0.51 (0.03)
23	α-gurjunene	1419	0.63 (0.04)	0.64 (0.04)	0.49 (0.03)	0.83 (0.04)
24	204[M+](25) 105(100) 91(92)	1423	0.41 (0.03)	0.34 (0.02)	0.49 (0.03)	0.34 (0.02)
25	(-)-aristolene	1427	0.25 (0.02)	0.38 (0.02)	0.24 (0.02)	0.38 (0.02)
26	γ-maaliene	1430	0.63 (0.04)	0.84 (0.04)	0.86 (0.04)	0.83 (0.05)
27	α-maaliene	1438	0.50 (0.03)	0.49 (0.03)	0.61 (0.04)	0.77 (0.04)
28	alloaromadendrene	1457	4.32 (0.05)	4.91 (0.06)	3.67 (0.07)	3.28 (0.08)
29	γ-gurjunene	1463	1.40 (0.04)	1.28 (0.05)	1.23 (0.09)	1.15 (0.06)
30	204[M+](13) 105(100) 91(85)	1469	0.50 (0.03)	0.77 (0.04)	0.74 (0.06)	0.64 (0.04)
31	204[M+](18) 91(100) 105(92)	1471	1.01 (0.04)	1.02 (0.05)	1.23 (0.09)	1.15 (0.04)
32	germacrene D	1474	0.50 (0.03)	0.64 (0.03)	0.61 (0.03)	0.51 (0.02)
33	ledene	1476	2.67 (0.07)	2.24 (0.06)	1.84 (0.07)	2.99 (0.08)
34	bicyclogermacrene	1488	35.02 (0.21)	33.83 (0.19)	34.78 (0.22)	33.10 (0.25)
35	γ-humulene	1493	2.02 (0.06)	1.59 (0.05)	2.24 (0.07)	2.03 (0.08)
36	cuparene	1502	3.91 (0.05)	4.03 (0.07)	3.99 (0.06)	3.60 (0.09)
37	α-bulnesene	1505	0.63 (0.04)	0.69 (0.04)	0.67 (0.04)	0.59 (0.03)
38	valencene	1510	-	-	-	-
39	4,5,9,10-dehydro-isolongifolene	1544	0.41 (0.03)	0.38 (0.02)	0.37 (0.02)	0.38 (0.03)
40	218[M+](5) 173(100) 145(97)	1555	0.39 (0.02)	0.51 (0.04)	0.49 (0.03)	0.70 (0.04)
41	spathulenol	1570	0.69 (0.04)	0.88 (0.03)	0.76 (0.04)	0.55 (0.03)
42	222[M+](5) 107(100) 147(75)	1573	0.63 (0.05)	0.64 (0.03)	0.74 (0.04)	0.64 (0.03)
43	220[M+](2) 159(100) 96(75)	1576	0.94 (0.05)	0.87 (0.04)	0.86 (0.04)	0.76 (0.04)
44	globulol	1590	0.76 (0.04)	0.64 (0.04)	0.61 (0.03)	0.64 (0.04)
45	220[M+](2) 120(100) 105(55)	1593	1.16 (0.06)	1.13 (0.06)	1.23 (0.05)	1.15 (0.05)
46	bisabola-2,10-diene 1,9-oxide	1602	0.71 (0.03)	0.63 (0.04)	0.61 (0.04)	0.77 (0.04)
47	220[M+](6) 119(100) 91(88)	1606	0.69 (0.04)	0.78 (0.03)	0.74 (0.04)	0.83 (0.05)
48	220[M+](2) 94(100) 109(85)	1613	1.42 (0.06)	1.15 (0.05)	1.36 (0.05)	1.15 (0.05)
49	ledene oxide-(II)	1629	4.79 (0.08)	4.52 (0.07)	4.70 (0.06)	3.99 (0.07)
50	isospathulenol	1631	-	-	-	-
51	α-acorenol	1633	0.38 (0.02)	0.25 (0.02)	0.37 (0.03)	0.25 (0.01)
52	234[M+](2) 105(100) 91(96)	1664	1.04 (0.04)	1.15 (0.05)	0.99 (0.06)	0.90 (0.03)
53	234[M+](3) 109(100) 91(85)	1672	0.71 (0.05)	0.89 (0.04)	0.74 (0.04)	0.64 (0.02)
54	aromadendrane-4,10-diol	1683	0.16 (0.01)	0.21 (0.01)	0.19 (0.02)	0.17 (0.01)

55	234[M+](4) 41(100) 109(98)	1686	0.24 (0.01)	0.20 (0.02)	0.22 (0.01)	0.20 (0.01)
56	232[M+](1) 105(100) 159(60)	1691	1.04 (0.06)	0.63 (0.03)	0.73 (0.03)	1.09 (0.04)
57	236[M+](1) 43(100) 139(65)	1694	2.92 (0.05)	2.43 (0.04)	2.85 (0.07)	2.68 (0.05)
58	1,4-dimethyl-7-isopropyl-azulene	1772	0.25 (0.02)	0.38 (0.03)	0.24 (0.02)	0.25 (0.01)
59	276[M+](1) 173(100) 109(50)	1805	0.14 (0.01)	0.13 (0.01)	0.12 (0.01)	0.11 (0.01)
60	276[M+] (8) 95(100) 43(66)	1818	0.19 (0.01)	0.19 (0.01)	0.23 (0.01)	0.23 (0.01)
61	280[M+](1) 110(100) 91(50)	1849	0.18 (0.01)	0.21 (0.02)	0.17 (0.01)	0.30 (0.02)
62	280[M+](1) 147(100) 119(70)	1924	0.13 (0.01)	0.15 (0.01)	0.08 (0.01)	0.12 (0.01)
63	geranyllinalool	2034	0.26 (0.02)	0.35 (0.02)	0.30 (0.01)	0.32 (0.03)
64	280[M+](2) 91(100) 105(85)	2041	-	-	-	-
65	280[M+](1) 91(100) 77(59)	2063	-	-	-	-
	Total		99.29 (2.31)	99.71 (2.23)	99.75 (2.42)	97.82 (2.27)
	% Identified		79.18 (1.53)	80.30 (1.51)	79.27 (1.60)	76.60 (1.59)
	Including the following:					
	Aliphatics		-	-	-	-
	Aromatics		2.47 (0.13)	2.75 (0.09)	3.19 (0.13)	2.50 (0.12)
	Monoterpene hydrocarbons		4.97 (0.22)	4.95 (0.24)	5.05 (0.20)	4.25 (0.16)
	Sesquiterpene hydrocarbons		63.99 (0.94)	65.12 (0.95)	63.49 (1.04)	63.16 (1.08)
	Sesquiterpenoide hydrocarbons		7.75 (0.24)	7.48 (0.23)	7.54 (0.23)	6.69 (0.23)

- less than 0.01%. [a] Retention index on Quadrex 007-5MS column. [b] For abbreviations of samples see Table S6. Mean % and ( ) standard deviation for sample analyzed in triplicate.

Table S1. (e) Volatile compounds detected in samples CSL-1 – CSL-3.

No.	Compounds	RI [a]	Code [b]		
			CSL-1	CSL-2	CSL-3
1	3-hydroxybutan-2-one	<700	0.20 (0.01)	0.25 (0.01)	0.25 (0.02)
2	3-methylbutan-1-ol	706	0.41 (0.02)	0.75 (0.03)	0.45 (0.02)
3	hexan-1-ol	867	-	-	-
4	tricyclene	926	0.10 (0.01)	0.12 (0.01)	0.12 (0.01)
5	α-pinene	936	0.10 (0.01)	0.18 (0.01)	0.15 (0.01)
6	2-methylpentan-2,4-diol	938	-	-	-
7	camphene	953	0.20 (0.01)	0.25 (0.01)	0.25 (0.01)
8	benzaldehyde	960	0.30 (0.02)	0.37 (0.02)	0.28 (0.01)
9	β-pinene	978	0.51 (0.02)	0.58 (0.03)	0.48 (0.03)
10	7-octen-4-ol	982	-	-	-
11	3-carene	1009	-	-	-
12	benzenemethanol	1023	1.90 (0.05)	2.12 (0.06)	2.05 (0.05)
13	120[M+](16) 91(100) 79(35)	1041	0.10 (0.01)	0.25 (0.01)	0.30 (0.02)
14	benzeneethanol	1114	0.70 (0.02)	0.75 (0.03)	0.63 (0.03)
15	β-cyclocitral	1222	-	-	-
16	phenoxyethanol	1226	0.20 (0.01)	0.27 (0.02)	0.18 (0.01)
17	189[M+](3) 121(100) 93(90)	1322	0.05 (0.01)	0.06 (0.01)	0.12 (0.01)
18	bicycloelemene	1325	0.70 (0.03)	1.10 (0.05)	1.20 (0.06)
19	202[M+](10) 96(100) 81(85)	1350	0.30 (0.02)	0.35 (0.03)	0.38 (0.02)
20	anastreptene	1370	14.53 (0.12)	15.04 (0.14)	12.83 (0.10)
21	α-ylangene	1373	0.50 (0.03)	0.25 (0.01)	0.30 (0.03)
22	β-elemene	1391	0.82 (0.05)	0.75 (0.03)	0.63 (0.04)
23	α-gurjunene	1419	0.30 (0.02)	0.33 (0.01)	0.29 (0.02)
24	204[M+](25) 105(100) 91(92)	1423	0.30 (0.02)	0.37 (0.02)	0.50 (0.03)
25	(-)-aristolene	1427	0.10 (0.01)	0.25 (0.01)	0.12 (0.01)
26	γ-maaliene	1430	0.20 (0.01)	0.37 (0.02)	0.28 (0.01)
27	α-maaliene	1438	0.30 (0.02)	0.27 (0.01)	0.38 (0.02)
28	alloaromadendrene	1457	3.10 (0.07)	2.90 (0.06)	3.04 (0.07)
29	γ-gurjunene	1463	0.72 (0.04)	0.62 (0.04)	0.50 (0.04)
30	204[M+](13) 105(100) 91(85)	1469	0.10 (0.01)	0.14 (0.01)	0.09 (0.01)
31	204[M+](18) 91(100) 105(92)	1471	0.30 (0.02)	0.37 (0.02)	0.41 (0.02)
32	germacrene D	1474	0.20 (0.01)	0.37 (0.03)	0.31 (0.02)
33	ledene	1476	1.40 (0.04)	1.90 (0.06)	1.77 (0.05)
34	bicyclogermacrene	1488	21.75 (0.13)	20.67 (0.18)	22.01 (0.16)
35	γ-humulene	1493	1.81 (0.06)	1.60 (0.04)	1.64 (0.04)
36	cuparene	1502	1.95 (0.05)	1.87 (0.05)	1.62 (0.05)
37	α-bulnesene	1505	0.82 (0.04)	0.80 (0.03)	0.88 (0.03)
38	valencene	1510	0.20 (0.01)	0.12 (0.01)	0.25 (0.01)
39	4,5,9,10-dehydro-isolongifolene	1544	0.20 (0.02)	0.27 (0.01)	0.19 (0.01)
40	218[M+](5) 173(100) 145(97)	1555	0.10 (0.01)	0.18 (0.01)	0.14 (0.01)
41	spathulenol	1570	0.40 (0.02)	0.31 (0.02)	0.42 (0.03)
42	222[M+](5) 107(100) 147(75)	1573	0.30 (0.01)	0.27 (0.01)	0.34 (0.02)
43	220[M+](2) 159(100) 96(75)	1576	0.20 (0.01)	0.28 (0.02)	0.24 (0.02)
44	globulol	1590	-	-	-
45	220[M+](2) 120(100) 105(55)	1593	2.13 (0.05)	2.40 (0.04)	2.68 (0.06)
46	bisabola-2,10-diene 1,9-oxide	1602	0.70 (0.04)	0.87 (0.04)	0.67 (0.04)
47	220[M+](6) 119(100) 91(88)	1606	0.20 (0.01)	0.25 (0.01)	0.34 (0.02)
48	220[M+](2) 94(100) 109(85)	1613	0.80 (0.03)	1.00 (0.04)	0.94 (0.03)
49	ledene oxide-(II)	1629	0.80 (0.03)	1.00 (0.05)	0.91 (0.02)
50	isospathulenol	1631	1.10 (0.06)	0.99 (0.04)	1.52 (0.04)
51	α-acorenol	1633	0.30 (0.02)	0.37 (0.02)	0.25 (0.01)
52	234[M+](2) 105(100) 91(96)	1664	0.30 (0.02)	0.37 (0.01)	0.27 (0.01)
53	234[M+](3) 109(100) 91(85)	1672	0.30 (0.03)	0.50 (0.03)	0.63 (0.05)
54	aromadendrane-4,10-diol	1683	1.10 (0.05)	1.80 (0.05)	1.60 (0.06)

55	234[M+](4) 41(100) 109(98)	1686	0.40 (0.02)	0.37 (0.01)	0.44 (0.03)
56	232[M+](1) 105(100) 159(60)	1691	-	-	-
57	236[M+](1) 43(100) 139(65)	1694	29.87 (0.17)	28.15 (0.21)	29.70 (0.19)
58	1,4-dimethyl-7-isopropyl-azulene	1772	0.72 (0.03)	0.80 (0.04)	0.90 (0.05)
59	276[M+](1) 173(100) 109(50)	1805	-	-	-
60	276[M+] (8) 95(100) 43(66)	1818	-	-	-
61	280[M+](1) 110(100) 91(50)	1849	2.40 (0.05)	2.63 (0.06)	2.48 (0.04)
62	280[M+](1) 147(100) 119(70)	1924	0.10 (0.01)	0.12 (0.01)	0.06 (0.01)
63	geranyllinalool	2034	0.10 (0.01)	0.20 (0.02)	0.25 (0.01)
64	280[M+](2) 91(100) 105(85)	2041	0.10 (0.01)	0.12 (0.01)	0.12 (0.01)
65	280[M+](1) 91(100) 77(59)	2063	-	-	-
	Total		97.79 (1.72)	99.64 (1.87)	99.78 (1.84)
	% Identified		59.44 (1.20)	61.46 (1.30)	59.60 (1.23)
	Including the following:				
	Aliphatics		0.61 (0.03)	1.00 (0.04)	0.70 (0.04)
	Aromatics		3.10 (0.10)	3.51 (0.13)	3.14 (0.10)
	Monoterpene hydrocarbons		0.91 (0.05)	1.13 (0.06)	1.00 (0.06)
	Sesquiterpene hydrocarbons		50.32 (0.79)	50.28 (0.83)	49.14 (0.82)
	Sesquiterpenoide hydrocarbons		4.50 (0.23)	5.54 (0.24)	5.62 (0.21)

- less than 0.01%. [a] Retention index on Quadrex 007-5MS column. [b] For abbreviations of samples see Table S7. Mean % and ( ) standard deviation for sample analyzed in triplicate.

Table S2. (a) Volatile compounds detected in samples CSS-9 – CSS-12.

No.	Compounds	RI [a]	Code [b]			
			CSS-9	CSS-10	CSS-11	CSS-12
1	3-hydroxybutan-2-one	<700	0.43 (0.03)	0.27 (0.01)	0.34 (0.02)	0.19 (0.01)
2	3-methylbutan-1-ol	706	0.18 (0.01)	0.36 (0.02)	0.22 (0.01)	0.17 (0.01)
3	hexan-1-ol	867	0.32 (0.02)	0.27 (0.01)	0.28 (0.02)	0.27 (0.02)
4	tricyclene	926	0.06 (0.01)	0.07 (0.01)	0.08 (0.01)	0.10 (0.01)
5	α-pinene	936	0.33 (0.03)	0.43 (0.03)	0.43 (0.03)	0.34 (0.02)
6	2-methylpentan-2,4-diol	938	0.35 (0.02)	0.36 (0.02)	0.43 (0.02)	0.26 (0.01)
7	camphene	953	0.51 (0.03)	0.48 (0.03)	0.82 (0.04)	0.64 (0.03)
8	benzaldehyde	960	0.99 (0.05)	0.87 (0.04)	1.21 (0.06)	1.02 (0.05)
9	β-pinene	978	0.94 (0.05)	1.23 (0.05)	0.97 (0.05)	0.73 (0.03)
10	7-octen-4-ol	982	0.43 (0.03)	0.26 (0.01)	0.32 (0.02)	0.29 (0.01)
11	3-carene	1009	-	-	-	-
12	benzenemethanol	1023	1.36 (0.06)	1.87 (0.06)	2.18 (0.08)	2.03 (0.06)
13	120[M+](16) 91(100) 79(35)	1041	1.17 (0.08)	0.98 (0.04)	0.82 (0.03)	0.81 (0.04)
14	benzeneethanol	1114	0.57 (0.04)	0.29 (0.01)	0.43 (0.02)	0.38 (0.03)
15	β-cyclocitral	1222	0.17 (0.01)	0.12 (0.01)	0.16 (0.01)	0.21 (0.01)
16	phenoxyethanol	1226	1.57 (0.06)	1.89 (0.06)	1.78 (0.05)	1.36 (0.04)
17	189[M+](3) 121(100) 93(90)	1322	0.57 (0.03)	0.42 (0.02)	0.35 (0.02)	0.32 (0.02)
18	bicycloelemene	1325	2.63 (0.09)	2.67 (0.05)	2.06 (0.06)	2.23 (0.04)
19	202[M+](10) 96(100) 81(85)	1350	4.73 (0.10)	4.27 (0.06)	4.45 (0.07)	4.63 (0.06)
20	anastreptene	1370	7.13 (0.12)	7.25 (0.05)	6.93 (0.06)	7.04 (0.08)
21	α-ylangene	1373	0.11 (0.01)	0.09 (0.01)	0.12 (0.01)	0.13 (0.01)
22	β-elemene	1391	0.64 (0.03)	0.56 (0.02)	0.62 (0.03)	0.54 (0.02)
23	α-gurjunene	1419	0.90 (0.04)	0.90 (0.03)	0.80 (0.03)	0.98 (0.03)
24	204[M+](25) 105(100) 91(92)	1423	0.53 (0.02)	0.69 (0.03)	0.92 (0.04)	0.76 (0.03)
25	(-)-aristolene	1427	0.38 (0.02)	0.27 (0.02)	0.47 (0.03)	0.68 (0.03)
26	γ-maaliene	1430	1.54 (0.06)	1.43 (0.05)	1.22 (0.06)	1.06 (0.04)
27	α-maaliene	1438	0.61 (0.03)	0.63 (0.03)	0.57 (0.03)	0.65 (0.02)
28	alloaromadendrene	1457	3.21 (0.06)	4.03 (0.06)	4.28 (0.05)	4.53 (0.06)
29	γ-gurjunene	1463	2.47 (0.04)	2.93 (0.08)	2.68 (0.06)	2.81 (0.06)
30	204[M+](13) 105(100) 91(85)	1469	0.22 (0.01)	0.13 (0.01)	0.13 (0.01)	0.18 (0.01)
31	204[M+](18) 91(100) 105(92)	1471	0.52 (0.03)	0.38 (0.02)	0.38 (0.02)	0.41 (0.03)
32	germacrene D	1474	0.48 (0.04)	0.28 (0.02)	0.47 (0.02)	0.38 (0.02)
33	ledene	1476	3.56 (0.09)	4.01 (0.08)	3.98 (0.06)	4.03 (0.05)
34	bicyclogermacrene	1488	25.96 (0.23)	25.57 (0.19)	24.98 (0.21)	25.30 (0.24)
35	γ-humulene	1493	0.64 (0.03)	0.67 (0.03)	0.82 (0.03)	0.78 (0.03)
36	cuparene	1502	1.59 (0.05)	1.56 (0.05)	1.55 (0.05)	1.78 (0.05)
37	α-bulnesene	1505	0.61 (0.02)	0.47 (0.03)	0.55 (0.03)	0.58 (0.03)
38	valencene	1510	0.57 (0.03)	0.57 (0.02)	0.49 (0.02)	0.52 (0.03)
39	4,5,9,10-dehydro-isolongifolene	1544	0.27 (0.01)	0.35 (0.03)	0.41 (0.03)	0.42 (0.02)
40	218[M+](5) 173(100) 145(97)	1555	0.76 (0.03)	0.73 (0.04)	0.68 (0.03)	0.87 (0.04)
41	spathulenol	1570	2.03 (0.07)	1.87 (0.06)	1.87 (0.05)	1.93 (0.05)
42	222[M+](5) 107(100) 147(75)	1573	1.23 (0.05)	0.97 (0.03)	1.21 (0.04)	1.19 (0.06)
43	220[M+](2) 159(100) 96(75)	1576	2.37 (0.08)	2.24 (0.07)	2.17 (0.06)	2.23 (0.06)
44	globulol	1590	1.43 (0.06)	1.64 (0.05)	1.38 (0.04)	1.46 (0.05)
45	220[M+](2) 120(100) 105(55)	1593	0.62 (0.03)	0.71 (0.03)	0.73 (0.03)	0.62 (0.04)
46	bisabola-2,10-diene 1,9-oxide	1602	1.52 (0.03)	1.45 (0.05)	1.23 (0.05)	1.78 (0.05)
47	220[M+](6) 119(100) 91(88)	1606	2.33 (0.05)	2.54 (0.06)	2.37 (0.06)	2.25 (0.07)
48	220[M+](2) 94(100) 109(85)	1613	1.67 (0.04)	1.43 (0.04)	1.97 (0.05)	1.73 (0.06)
49	ledene oxide-(II)	1629	2.97 (0.06)	2.57 (0.05)	2.68 (0.07)	2.87 (0.08)
50	isospathulenol	1631	2.84 (0.04)	2.97 (0.06)	2.54 (0.06)	2.57 (0.09)
51	α-acorenol	1633	0.67 (0.03)	0.57 (0.03)	0.49 (0.03)	0.57 (0.03)
52	234[M+](2) 105(100) 91(96)	1664	0.37 (0.02)	0.39 (0.02)	0.44 (0.02)	0.39 (0.02)
53	234[M+](3) 109(100) 91(85)	1672	0.77 (0.03)	0.99 (0.03)	0.98 (0.04)	0.87 (0.04)
54	aromadendrane-4,10-diol	1683	0.33 (0.02)	0.37 (0.01)	0.35 (0.01)	0.41 (0.03)

55	234[M+](4) 41(100) 109(98)	1686	0.79 (0.04)	0.76 (0.04)	0.87 (0.03)	0.92 (0.04)
56	232[M+](1) 105(100) 159(60)	1691	1.21 (0.05)	1.47 (0.06)	1.64 (0.05)	1.43 (0.05)
57	236[M+](1) 43(100) 139(65)	1694	1.07 (0.03)	1.09 (0.03)	1.47 (0.04)	1.41 (0.06)
58	1,4-dimethyl-7-isopropyl-azulene	1772	0.37 (0.01)	0.29 (0.01)	0.28 (0.01)	0.33 (0.02)
59	276[M+](1) 173(100) 109(50)	1805	2.86 (0.10)	2.31 (0.06)	2.54 (0.06)	2.23 (0.06)
60	276[M+] (8) 95(100) 43(66)	1818	0.36 (0.02)	0.51 (0.01)	0.32 (0.02)	0.34 (0.02)
61	280[M+](1) 110(100) 91(50)	1849	0.47 (0.03)	0.41 (0.02)	0.39 (0.03)	0.34 (0.03)
62	280[M+](1) 147(100) 119(70)	1924	0.41 (0.03)	0.36 (0.02)	0.29 (0.01)	0.27 (0.02)
63	geranyllinalool	2034	0.13 (0.01)	0.18 (0.01)	0.11 (0.01)	0.13 (0.01)
64	280[M+](2) 91(100) 105(85)	2041	0.18 (0.01)	0.37 (0.02)	0.39 (0.02)	0.29 (0.02)
65	280[M+](1) 91(100) 77(59)	2063	0.18 (0.01)	0.19 (0.01)	0.14 (0.01)	0.13 (0.01)
	Total		99.19 (2.70)	99.26 (2.32)	99.23 (2.43)	99.10 (2.50)
	% Identified		73.80 (1.78)	74.92 (1.55)	73.58 (1.64)	74.48 (1.61)
	Including the following:					
	Aliphatics		1.71 (0.11)	1.52 (0.07)	1.59 (0.09)	1.18 (0.06)
	Aromatics		4.49 (0.21)	4.92 (0.17)	5.60 (0.21)	4.79 (0.18)
	Monoterpene hydrocarbons		2.01 (0.13)	2.33 (0.13)	2.46 (0.14)	2.02 (0.10)
	Sesquiterpene hydrocarbons		53.67 (1.01)	54.53 (0.86)	53.28 (0.88)	54.77 (0.88)
	Sesquiterpenoide hydrocarbons		11.92 (0.32)	11.62 (0.32)	10.65 (0.32)	11.72 (0.39)

- less than 0.01%. [a] Retention index on Quadrex 007-5MS column. [b] For abbreviations of samples see Table S8. Mean % and ( ) standard deviation for sample analyzed in triplicate.

Table S2. (b) Volatile compounds detected in samples CSS-13 – CSS-16.

No.	Compounds	RI [a]	Code [b]			
			CSS-13	CSS-14	CSS-15	CSS-16
1	3-hydroxybutan-2-one	<700	0.22 (0.01)	0.18 (0.01)	0.42 (0.03)	0.36 (0.02)
2	3-methylbutan-1-ol	706	0.27 (0.01)	0.12 (0.01)	0.17 (0.01)	0.22 (0.01)
3	hexan-1-ol	867	0.37 (0.02)	0.33 (0.02)	0.26 (0.01)	0.29 (0.01)
4	tricyclene	926	0.06 (0.01)	0.03 (0.01)	0.04 (0.01)	0.05 (0.01)
5	α-pinene	936	0.27 (0.02)	0.38 (0.02)	0.37 (0.02)	0.34 (0.02)
6	2-methylpentan-2,4-diol	938	0.28 (0.01)	0.39 (0.02)	0.52 (0.03)	0.48 (0.03)
7	camphene	953	0.53 (0.03)	0.36 (0.02)	0.54 (0.02)	0.63 (0.03)
8	benzaldehyde	960	0.98 (0.03)	1.33 (0.04)	1.01 (0.05)	0.96 (0.03)
9	β-pinene	978	0.86 (0.02)	1.03 (0.04)	1.09 (0.04)	1.12 (0.04)
10	7-octen-4-ol	982	0.36 (0.02)	0.28 (0.01)	0.28 (0.01)	0.35 (0.02)
11	3-carene	1009	-	-	-	-
12	benzenemethanol	1023	1.96 (0.05)	3.05 (0.04)	1.73 (0.06)	1.65 (0.05)
13	120[M+](16) 91(100) 79(35)	1041	0.86 (0.03)	1.24 (0.03)	1.09 (0.05)	1.03 (0.04)
14	benzeneethanol	1114	0.34 (0.02)	0.64 (0.03)	0.36 (0.02)	0.41 (0.03)
15	β-cyclocitral	1222	0.19 (0.01)	0.38 (0.02)	0.11 (0.01)	0.16 (0.01)
16	phenoxyethanol	1226	1.83 (0.05)	1.25 (0.04)	1.64 (0.04)	2.03 (0.06)
17	189[M+](3) 121(100) 93(90)	1322	0.38 (0.02)	0.28 (0.01)	0.53 (0.03)	0.51 (0.02)
18	bicycloelemene	1325	2.97 (0.06)	1.79 (0.05)	2.34 (0.03)	2.28 (0.05)
19	202[M+](10) 96(100) 81(85)	1350	4.39 (0.06)	4.69 (0.04)	4.18 (0.05)	4.56 (0.07)
20	anastreptene	1370	6.87 (0.07)	5.68 (0.06)	6.93 (0.06)	6.18 (0.09)
21	α-ylangene	1373	0.10 (0.01)	0.31 (0.01)	0.14 (0.01)	0.12 (0.01)
22	β-elemene	1391	0.57 (0.02)	0.55 (0.02)	0.59 (0.02)	0.63 (0.03)
23	α-gurjunene	1419	1.05 (0.03)	0.94 (0.02)	0.87 (0.03)	0.76 (0.04)
24	204[M+](25) 105(100) 91(92)	1423	0.89 (0.03)	0.79 (0.03)	0.73 (0.03)	0.54 (0.03)
25	(-)-aristolene	1427	0.52 (0.02)	0.37 (0.02)	0.33 (0.02)	0.42 (0.02)
26	γ-maaliene	1430	1.73 (0.04)	0.98 (0.03)	1.27 (0.04)	1.18 (0.06)
27	α-maaliene	1438	0.48 (0.02)	0.61 (0.02)	0.54 (0.03)	0.55 (0.04)
28	alloaromadendrene	1457	4.39 (0.05)	3.48 (0.06)	3.78 (0.05)	3.64 (0.08)
29	γ-gurjunene	1463	2.87 (0.04)	2.91 (0.04)	2.97 (0.04)	2.43 (0.06)
30	204[M+](13) 105(100) 91(85)	1469	0.23 (0.01)	0.28 (0.02)	0.18 (0.01)	0.23 (0.01)
31	204[M+](18) 91(100) 105(92)	1471	0.45 (0.02)	0.61 (0.03)	0.45 (0.02)	0.41 (0.02)
32	germacrene D	1474	0.37 (0.02)	0.42 (0.02)	0.52 (0.02)	0.41 (0.02)
33	ledene	1476	4.15 (0.03)	3.21 (0.06)	3.39 (0.03)	3.29 (0.04)
34	bicyclogermacrene	1488	25.04 (0.23)	24.25 (0.25)	26.78 (0.18)	25.63 (0.26)
35	γ-humulene	1493	0.85 (0.03)	1.02 (0.04)	0.84 (0.04)	0.78 (0.04)
36	cuparene	1502	1.73 (0.06)	1.42 (0.04)	1.44 (0.06)	1.46 (0.06)
37	α-bulnesene	1505	0.52 (0.03)	0.53 (0.02)	0.57 (0.03)	0.59 (0.03)
38	valencene	1510	0.49 (0.03)	0.33 (0.02)	0.61 (0.03)	0.63 (0.03)
39	4,5,9,10-dehydro-isolongifolene	1544	0.28 (0.01)	0.86 (0.03)	0.43 (0.04)	0.34 (0.01)
40	218[M+](5) 173(100) 145(97)	1555	0.89 (0.02)	1.15 (0.04)	0.65 (0.03)	0.67 (0.02)
41	spathulenol	1570	1.90 (0.05)	1.98 (0.05)	1.93 (0.06)	1.99 (0.05)
42	222[M+](5) 107(100) 147(75)	1573	1.38 (0.04)	1.41 (0.04)	1.22 (0.05)	1.18 (0.06)
43	220[M+](2) 159(100) 96(75)	1576	2.17 (0.05)	3.01 (0.06)	2.19 (0.07)	2.47 (0.07)
44	globulol	1590	1.67 (0.04)	1.53 (0.03)	1.72 (0.05)	1.69 (0.04)
45	220[M+](2) 120(100) 105(55)	1593	0.59 (0.03)	0.87 (0.02)	0.78 (0.03)	0.67 (0.04)
46	bisabola-2,10-diene 1,9-oxide	1602	1.76 (0.04)	1.52 (0.04)	1.53 (0.04)	1.47 (0.03)
47	220[M+](6) 119(100) 91(88)	1606	2.31 (0.05)	3.05 (0.04)	2.44 (0.05)	2.78 (0.05)
48	220[M+](2) 94(100) 109(85)	1613	1.54 (0.04)	2.07 (0.04)	1.67 (0.04)	1.72 (0.04)
49	ledene oxide-(II)	1629	2.57 (0.05)	2.88 (0.05)	2.63 (0.06)	2.67 (0.06)
50	isospathulenol	1631	2.54 (0.05)	2.33 (0.03)	2.83 (0.05)	2.94 (0.05)
51	α-acorenol	1633	0.46 (0.02)	0.62 (0.02)	0.59 (0.04)	0.56 (0.03)
52	234[M+](2) 105(100) 91(96)	1664	0.34 (0.02)	0.78 (0.03)	0.29 (0.01)	0.55 (0.02)
53	234[M+](3) 109(100) 91(85)	1672	0.96 (0.03)	0.67 (0.03)	0.83 (0.02)	0.85 (0.03)
54	aromadendrane-4,10-diol	1683	0.46 (0.02)	0.42 (0.02)	0.36 (0.01)	0.39 (0.01)

55	234[M+](4) 41(100) 109(98)	1686	0.86 (0.03)	0.65 (0.03)	0.67 (0.03)	0.74 (0.03)
56	232[M+](1) 105(100) 159(60)	1691	1.62 (0.04)	1.73 (0.05)	1.54 (0.04)	1.62 (0.06)
57	236[M+](1) 43(100) 139(65)	1694	1.01 (0.03)	1.38 (0.04)	1.16 (0.03)	1.21 (0.04)
58	1,4-dimethyl-7-isopropyl-azulene	1772	0.34 (0.01)	0.25 (0.02)	0.27 (0.01)	0.34 (0.02)
59	276[M+](1) 173(100) 109(50)	1805	1.98 (0.05)	1.78 (0.05)	2.27 (0.06)	2.31 (0.05)
60	276[M+] (8) 95(100) 43(66)	1818	0.27 (0.01)	0.35 (0.01)	0.34 (0.02)	0.34 (0.02)
61	280[M+](1) 110(100) 91(50)	1849	0.36 (0.02)	0.37 (0.02)	0.38 (0.01)	0.44 (0.03)
62	280[M+](1) 147(100) 119(70)	1924	0.26 (0.01)	0.37 (0.01)	0.24 (0.01)	0.36 (0.02)
63	geranyllinalool	2034	0.14 (0.01)	0.09 (0.01)	0.21 (0.01)	0.16 (0.01)
64	280[M+](2) 91(100) 105(85)	2041	0.44 (0.02)	0.26 (0.02)	0.17 (0.01)	0.14 (0.01)
65	280[M+](1) 91(100) 77(59)	2063	0.11 (0.01)	0.10 (0.01)	0.22 (0.01)	0.23 (0.01)
	Total		99.63 (2.07)	98.92 (2.11)	99.17 (2.16)	98.14 (2.43)
	% Identified		75.34 (1.40)	71.03 (1.41)	74.95 (1.45)	72.58 (1.64)
	Including the following:					
	Aliphatics		1.50 (0.07)	1.30 (0.07)	1.65 (0.09)	1.70 (0.09)
	Aromatics		5.11 (0.15)	6.27 (0.15)	4.74 (0.17)	5.05 (0.17)
	Monoterpene hydrocarbons		1.91 (0.09)	2.18 (0.11)	2.15 (0.10)	2.30 (0.11)
	Sesquiterpene hydrocarbons		55.32 (0.81)	49.91 (0.83)	54.61 (0.77)	51.66 (0.99)
	Sesquiterpenoide hydrocarbons		11.50 (0.28)	11.37 (0.25)	11.80 (0.32)	11.87 (0.28)

- less than 0.01%. [a] Retention index on Quadrex 007-5MS column. [b] For abbreviations of samples see Table S8. Mean % and ( ) standard deviation for sample analyzed in triplicate.

Table S2. (c) Volatile compounds detected in samples CSP-9 – CSP-12.

No.	Compounds	RI [a]	Code [b]			
			CSP-9	CSP-10	CSP-11	CSP-12
1	3-hydroxybutan-2-one	<700	-	-	-	-
2	3-methylbutan-1-ol	706	-	-	-	-
3	hexan-1-ol	867	-	-	-	-
4	tricyclene	926	0.33 (0.02)	0.45 (0.03)	0.24 (0.02)	0.25 (0.02)
5	α-pinene	936	1.63 (0.05)	1.63 (0.05)	1.55 (0.05)	1.38 (0.04)
6	2-methylpentan-2,4-diol	938	-	-	-	-
7	camphene	953	0.83 (0.03)	0.67 (0.03)	0.79 (0.04)	0.85 (0.03)
8	benzaldehyde	960	0.28 (0.01)	0.25 (0.01)	0.21 (0.02)	0.26 (0.02)
9	β-pinene	978	1.47 (0.06)	1.64 (0.05)	1.75 (0.06)	1.63 (0.05)
10	7-octen-4-ol	982	-	-	-	-
11	3-carene	1009	0.26 (0.01)	0.37 (0.02)	0.23 (0.02)	0.27 (0.02)
12	benzenemethanol	1023	1.30 (0.05)	1.01 (0.04)	1.30 (0.06)	1.17 (0.06)
13	120[M+](16) 91(100) 79(35)	1041	1.07 (0.04)	0.94 (0.04)	0.98 (0.05)	1.11 (0.05)
14	benzeneethanol	1114	0.08 (0.01)	0.09 (0.01)	0.08 (0.01)	0.11 (0.01)
15	β-cyclocitral	1222	0.14 (0.01)	0.09 (0.01)	0.21 (0.02)	0.13 (0.01)
16	phenoxyethanol	1226	0.71 (0.02)	0.78 (0.03)	0.83 (0.04)	0.74 (0.04)
17	189[M+](3) 121(100) 93(90)	1322	0.12 (0.01)	0.09 (0.01)	0.21 (0.01)	0.17 (0.02)
18	bicycloelemene	1325	1.43 (0.05)	1.33 (0.06)	1.28 (0.07)	1.18 (0.05)
19	202[M+](10) 96(100) 81(85)	1350	5.23 (0.07)	4.96 (0.09)	4.97 (0.10)	5.04 (0.08)
20	anastreptene	1370	9.21 (0.09)	8.97 (0.12)	9.44 (0.12)	9.57 (0.11)
21	α-ylangene	1373	0.41 (0.03)	0.42 (0.03)	0.31 (0.02)	0.27 (0.03)
22	β-elemene	1391	0.53 (0.03)	0.63 (0.03)	0.47 (0.03)	0.59 (0.04)
23	α-gurjunene	1419	0.59 (0.03)	0.69 (0.04)	0.73 (0.04)	0.63 (0.04)
24	204[M+](25) 105(100) 91(92)	1423	0.29 (0.01)	0.37 (0.02)	0.41 (0.03)	0.38 (0.03)
25	(-)-aristolene	1427	0.35 (0.02)	0.19 (0.01)	0.36 (0.02)	0.33 (0.02)
26	γ-maaliene	1430	0.71 (0.03)	0.87 (0.04)	0.74 (0.04)	0.61 (0.03)
27	α-maaliene	1438	0.37 (0.02)	0.61 (0.03)	0.43 (0.03)	0.45 (0.02)
28	alloaromadendrene	1457	4.97 (0.04)	4.69 (0.06)	5.01 (0.07)	4.78 (0.06)
29	γ-gurjunene	1463	1.47 (0.03)	1.39 (0.04)	1.32 (0.05)	1.44 (0.04)
30	204[M+](13) 105(100) 91(85)	1469	0.54 (0.03)	0.61 (0.03)	0.62 (0.03)	0.54 (0.03)
31	204[M+](18) 91(100) 105(92)	1471	1.12 (0.05)	0.93 (0.04)	1.05 (0.04)	0.98 (0.05)
32	germacrene D	1474	0.57 (0.03)	0.49 (0.03)	0.62 (0.04)	0.55 (0.03)
33	ledene	1476	2.31 (0.06)	2.79 (0.06)	2.41 (0.07)	2.39 (0.06)
34	bicyclogermacrene	1488	31.96 (0.27)	34.27 (0.21)	33.21 (0.26)	32.97 (0.24)
35	γ-humulene	1493	2.39 (0.05)	2.09 (0.05)	2.05 (0.08)	1.97 (0.06)
36	cuparene	1502	4.59 (0.04)	4.31 (0.07)	4.48 (0.11)	4.61 (0.09)
37	α-bulnesene	1505	0.64 (0.03)	0.68 (0.03)	0.71 (0.04)	0.67 (0.05)
38	valencene	1510	-	-	-	-
39	4,5,9,10-dehydro-isolongifolene	1544	0.46 (0.04)	0.53 (0.04)	0.38 (0.04)	0.42 (0.03)
40	218[M+](5) 173(100) 145(97)	1555	0.39 (0.03)	0.57 (0.03)	0.51 (0.02)	0.42 (0.03)
41	spathulenol	1570	0.86 (0.05)	0.64 (0.04)	0.67 (0.04)	0.48 (0.04)
42	222[M+](5) 107(100) 147(75)	1573	0.55 (0.03)	0.68 (0.03)	0.65 (0.05)	0.73 (0.05)
43	220[M+](2) 159(100) 96(75)	1576	0.85 (0.04)	0.96 (0.05)	0.99 (0.05)	0.87 (0.04)
44	globulol	1590	0.49 (0.03)	0.57 (0.03)	0.64 (0.04)	0.62 (0.04)
45	220[M+](2) 120(100) 105(55)	1593	1.17 (0.06)	1.27 (0.05)	1.15 (0.06)	1.28 (0.06)
46	bisabola-2,10-diene 1,9-oxide	1602	0.57 (0.03)	0.58 (0.03)	0.78 (0.04)	0.50 (0.04)
47	220[M+](6) 119(100) 91(88)	1606	0.70 (0.04)	0.68 (0.03)	0.85 (0.04)	0.69 (0.03)
48	220[M+](2) 94(100) 109(85)	1613	1.36 (0.05)	1.24 (0.04)	1.14 (0.05)	1.11 (0.05)
49	ledene oxide-(II)	1629	4.19 (0.11)	4.56 (0.08)	3.97 (0.08)	4.97 (0.07)
50	isospathulenol	1631	-	-	-	-
51	α-acorenol	1633	0.39 (0.03)	0.36 (0.02)	0.41 (0.02)	0.27 (0.03)
52	234[M+](2) 105(100) 91(96)	1664	0.99 (0.04)	1.21 (0.04)	0.97 (0.05)	1.15 (0.06)
53	234[M+](3) 109(100) 91(85)	1672	0.89 (0.03)	0.77 (0.03)	0.75 (0.04)	0.69 (0.04)
54	aromadendrane-4,10-diol	1683	0.32 (0.02)	0.23 (0.01)	0.27 (0.01)	0.23 (0.01)

55	234[M+](4) 41(100) 109(98)	1686	0.27 (0.02)	0.19 (0.01)	0.21 (0.02)	0.32 (0.03)
56	232[M+](1) 105(100) 159(60)	1691	0.87 (0.04)	1.03 (0.06)	1.07 (0.05)	0.97 (0.04)
57	236[M+](1) 43(100) 139(65)	1694	3.11 (0.09)	2.90 (0.07)	3.01 (0.08)	2.87 (0.09)
58	1,4-dimethyl-7-isopropyl-azulene	1772	0.41 (0.03)	0.42 (0.02)	0.24 (0.02)	0.29 (0.03)
59	276[M+](1) 173(100) 109(50)	1805	0.09 (0.01)	0.14 (0.01)	0.08 (0.01)	0.10 (0.01)
60	276[M+] (8) 95(100) 43(66)	1818	0.23 (0.01)	0.32 (0.01)	0.21 (0.01)	0.18 (0.01)
61	280[M+](1) 110(100) 91(50)	1849	0.24 (0.01)	0.13 (0.01)	0.16 (0.01)	0.22 (0.02)
62	280[M+](1) 147(100) 119(70)	1924	0.10 (0.01)	0.07 (0.01)	0.12 (0.02)	0.10 (0.02)
63	geranyllinalool	2034	0.41 (0.03)	0.29 (0.01)	0.41 (0.02)	0.33 (0.03)
64	280[M+](2) 91(100) 105(85)	2041	-	-	-	-
65	280[M+](1) 91(100) 77(59)	2063	-	-	-	-
	Total		97.81 (2.21)	99.64 (2.18)	98.64 (2.56)	97.83 (2.43)
	% Identified		77.63 (1.49)	79.58 (1.47)	78.53 (1.74)	77.91 (1.59)
	Including the following:					
	Aliphatics		-	-	-	-
	Aromatics		2.37 (0.09)	2.13 (0.09)	2.42 (0.13)	2.28 (0.13)
	Monoterpene hydrocarbons		4.66 (0.18)	4.85 (0.19)	4.77 (0.21)	4.51 (0.17)
	Sesquiterpene hydrocarbons		63.37 (0.92)	65.37 (0.97)	64.19 (1.15)	63.72 (1.03)
	Sesquiterpenoide hydrocarbons		7.23 (0.30)	7.23 (0.22)	7.15 (0.25)	7.40 (0.26)

- less than 0.01%. [a] Retention index on Quadrex 007-5MS column. [b] For abbreviations of samples see Table S9. Mean % and ( ) standard deviation for sample analyzed in triplicate.

Table S2. (d) Volatile compounds detected in samples CSP-13 – CSP-16.

No.	Compounds	RI [a]	Code [b]			
			CSP-13	CSP-14	CSP-15	CSP-16
1	3-hydroxybutan-2-one	<700	-	-	-	-
2	3-methylbutan-1-ol	706	-	-	-	-
3	hexan-1-ol	867	-	-	-	-
4	tricyclene	926	0.37 (0.03)	0.42 (0.02)	0.39 (0.02)	0.36 (0.02)
5	α-pinene	936	1.57 (0.04)	1.59 (0.04)	1.47 (0.05)	1.48 (0.06)
6	2-methylpentan-2,4-diol	938	-	-	-	-
7	camphene	953	0.88 (0.03)	0.93 (0.03)	0.92 (0.03)	0.84 (0.04)
8	benzaldehyde	960	0.26 (0.02)	0.34 (0.02)	0.27 (0.02)	0.33 (0.02)
9	β-pinene	978	1.66 (0.05)	1.57 (0.06)	1.54 (0.05)	1.34 (0.05)
10	7-octen-4-ol	982	-	-	-	-
11	3-carene	1009	0.29 (0.01)	0.33 (0.03)	0.25 (0.02)	0.23 (0.01)
12	benzenemethanol	1023	1.14 (0.06)	1.72 (0.05)	1.50 (0.06)	1.23 (0.06)
13	120[M+](16) 91(100) 79(35)	1041	0.96 (0.04)	0.99 (0.04)	1.09 (0.04)	1.21 (0.05)
14	benzeneethanol	1114	0.15 (0.01)	0.16 (0.01)	0.09 (0.01)	0.12 (0.01)
15	β-cyclocitral	1222	0.16 (0.01)	0.11 (0.01)	0.17 (0.01)	0.18 (0.01)
16	phenoxyethanol	1226	0.69 (0.04)	0.84 (0.04)	0.69 (0.03)	0.68 (0.04)
17	189[M+](3) 121(100) 93(90)	1322	0.08 (0.01)	0.13 (0.01)	0.18 (0.01)	0.11 (0.01)
18	bicycloelemene	1325	1.37 (0.04)	1.29 (0.05)	1.68 (0.06)	1.27 (0.06)
19	202[M+](10) 96(100) 81(85)	1350	5.14 (0.06)	5.22 (0.07)	5.17 (0.13)	5.19 (0.09)
20	anastreptene	1370	9.37 (0.08)	9.12 (0.11)	9.63 (0.15)	9.34 (0.13)
21	α-ylangene	1373	0.36 (0.02)	0.39 (0.03)	0.33 (0.02)	0.29 (0.02)
22	β-elemene	1391	0.49 (0.03)	0.45 (0.03)	0.61 (0.04)	0.54 (0.03)
23	α-gurjunene	1419	0.53 (0.03)	0.64 (0.04)	0.67 (0.05)	0.73 (0.06)
24	204[M+](25) 105(100) 91(92)	1423	0.43 (0.02)	0.52 (0.04)	0.37 (0.03)	0.46 (0.03)
25	(-)-aristolene	1427	0.27 (0.01)	0.31 (0.02)	0.28 (0.02)	0.36 (0.02)
26	γ-maaliene	1430	0.75 (0.04)	0.94 (0.07)	0.69 (0.04)	0.67 (0.05)
27	α-maaliene	1438	0.47 (0.03)	0.53 (0.04)	0.54 (0.05)	0.69 (0.06)
28	alloaromadendrene	1457	4.86 (0.06)	4.77 (0.12)	4.83 (0.09)	4.95 (0.10)
29	γ-gurjunene	1463	1.56 (0.04)	1.37 (0.06)	1.32 (0.05)	1.41 (0.05)
30	204[M+](13) 105(100) 91(85)	1469	0.63 (0.03)	0.71 (0.04)	0.78 (0.04)	0.73 (0.04)
31	204[M+](18) 91(100) 105(92)	1471	1.00 (0.05)	1.07 (0.05)	0.97 (0.05)	1.23 (0.05)
32	germacrene D	1474	0.47 (0.02)	0.53 (0.03)	0.61 (0.04)	0.53 (0.04)
33	ledene	1476	2.54 (0.06)	2.26 (0.07)	2.27 (0.06)	2.84 (0.07)
34	bicyclogermacrene	1488	34.01 (0.24)	32.87 (0.26)	33.27 (0.29)	33.28 (0.25)
35	γ-humulene	1493	2.18 (0.07)	2.37 (0.06)	2.21 (0.06)	1.99 (0.05)
36	cuparene	1502	4.01 (0.13)	4.20 (0.07)	4.11 (0.09)	4.08 (0.07)
37	α-bulnesene	1505	0.73 (0.05)	0.65 (0.04)	0.57 (0.04)	0.78 (0.03)
38	valencene	1510	-	-	-	-
39	4,5,9,10-dehydro-isolongifolene	1544	0.39 (0.02)	0.41 (0.03)	0.54 (0.05)	0.47 (0.03)
40	218[M+](5) 173(100) 145(97)	1555	0.44 (0.02)	0.43 (0.02)	0.47 (0.04)	0.61 (0.04)
41	spathulenol	1570	0.73 (0.04)	0.75 (0.05)	0.92 (0.07)	0.47 (0.03)
42	222[M+](5) 107(100) 147(75)	1573	0.67 (0.03)	0.71 (0.04)	0.86 (0.08)	0.77 (0.05)
43	220[M+](2) 159(100) 96(75)	1576	0.93 (0.04)	0.78 (0.04)	0.94 (0.07)	0.73 (0.04)
44	globulol	1590	0.54 (0.03)	0.64 (0.05)	0.73 (0.06)	0.76 (0.05)
45	220[M+](2) 120(100) 105(55)	1593	1.26 (0.03)	1.32 (0.07)	1.21 (0.06)	1.09 (0.06)
46	bisabola-2,10-diene 1,9-oxide	1602	0.73 (0.02)	0.64 (0.04)	0.61 (0.04)	0.47 (0.03)
47	220[M+](6) 119(100) 91(88)	1606	0.67 (0.03)	0.77 (0.05)	0.71 (0.05)	0.89 (0.04)
48	220[M+](2) 94(100) 109(85)	1613	1.53 (0.04)	1.47 (0.06)	1.23 (0.06)	1.13 (0.07)
49	ledene oxide-(II)	1629	4.67 (0.06)	4.89 (0.09)	4.26 (0.09)	4.08 (0.11)
50	isospathulenol	1631	-	-	-	-
51	α-acorenol	1633	0.43 (0.02)	0.40 (0.03)	0.31 (0.02)	0.28 (0.03)
52	234[M+](2) 105(100) 91(96)	1664	1.11 (0.05)	0.93 (0.04)	1.06 (0.04)	1.17 (0.05)
53	234[M+](3) 109(100) 91(85)	1672	0.69 (0.03)	0.63 (0.03)	0.73 (0.05)	0.86 (0.03)
54	aromadendrane-4,10-diol	1683	0.19 (0.02)	0.22 (0.01)	0.17 (0.02)	0.18 (0.01)

55	234[M+](4) 41(100) 109(98)	1686	0.19 (0.01)	0.27 (0.02)	0.30 (0.03)	0.23 (0.01)
56	232[M+](1) 105(100) 159(60)	1691	0.99 (0.06)	0.87 (0.04)	0.91 (0.04)	1.11 (0.05)
57	236[M+](1) 43(100) 139(65)	1694	2.88 (0.09)	2.94 (0.06)	2.99 (0.07)	2.86 (0.06)
58	1,4-dimethyl-7-isopropyl-azulene	1772	0.28 (0.02)	0.37 (0.02)	0.31 (0.02)	0.19 (0.02)
59	276[M+](1) 173(100) 109(50)	1805	0.11 (0.01)	0.13 (0.01)	0.18 (0.01)	0.10 (0.01)
60	276[M+] (8) 95(100) 43(66)	1818	0.15 (0.01)	0.27 (0.03)	0.19 (0.01)	0.27 (0.02)
61	280[M+](1) 110(100) 91(50)	1849	0.19 (0.01)	0.18 (0.01)	0.27 (0.02)	0.31 (0.03)
62	280[M+](1) 147(100) 119(70)	1924	0.12 (0.01)	0.09 (0.01)	0.18 (0.01)	0.13 (0.01)
63	geranyllinalool	2034	0.39 (0.03)	0.31 (0.02)	0.37 (0.03)	0.34 (0.02)
64	280[M+](2) 91(100) 105(85)	2041	-	-	-	-
65	280[M+](1) 91(100) 77(59)	2063	-	-	-	-
	Total		99.66 (2.19)	99.76 (2.53)	99.92 (2.79)	98.97 (2.58)
	% Identified		79.49 (1.51)	79.33 (1.75)	79.13 (1.85)	77.78 (1.74)
	Including the following:					
	Aliphatics		-	-	-	-
	Aromatics		2.24 (0.13)	3.06 (0.12)	2.55 (0.12)	2.36 (0.13)
	Monoterpene hydrocarbons		4.93 (0.17)	4.95 (0.19)	4.74 (0.18)	4.43 (0.19)
	Sesquiterpene hydrocarbons		64.64 (0.99)	63.47 (1.15)	64.47 (1.22)	64.41 (1.14)
	Sesquiterpenoide hydrocarbons		7.68 (0.22)	7.85 (0.29)	7.37 (0.33)	6.58 (0.28)

- less than 0.01%. [a] Retention index on Quadrex 007-5MS column. [b] For abbreviations of samples see Table S9. Mean % and ( ) standard deviation for sample analyzed in triplicate.

Table S2. (e) Volatile compounds detected in samples CSL-4 – CSL-6.

No.	Compounds	RI [a]	Code [b]		
			CSL-4	CSL-5	CSL-6
1	3-hydroxybutan-2-one	<700	0.24 (0.01)	0.27 (0.02)	0.18 (0.01)
2	3-methylbutan-1-ol	706	0.55 (0.03)	0.47 (0.03)	0.52 (0.03)
3	hexan-1-ol	867	-	-	-
4	tricyclene	926	0.15 (0.01)	0.11 (0.01)	0.09 (0.01)
5	α-pinene	936	0.14 (0.01)	0.12 (0.01)	0.10 (0.01)
6	2-methylpentan-2,4-diol	938	-	-	-
7	camphene	953	0.27 (0.02)	0.24 (0.01)	0.22 (0.01)
8	benzaldehyde	960	0.37 (0.02)	0.44 (0.03)	0.33 (0.02)
9	β-pinene	978	0.41 (0.03)	0.48 (0.03)	0.53 (0.03)
10	7-octen-4-ol	982	-	-	-
11	3-carene	1009	-	-	-
12	benzenemethanol	1023	2.14 (0.06)	2.01 (0.07)	1.97 (0.06)
13	120[M+](16) 91(100) 79(35)	1041	0.19 (0.01)	0.20 (0.01)	0.15 (0.01)
14	benzeneethanol	1114	0.63 (0.04)	0.71 (0.04)	0.69 (0.03)
15	β-cyclocitral	1222	-	-	-
16	phenoxyethanol	1226	0.18 (0.01)	0.22 (0.01)	0.29 (0.02)
17	189[M+](3) 121(100) 93(90)	1322	0.11 (0.01)	0.09 (0.01)	0.07 (0.01)
18	bicycloelemene	1325	1.09 (0.05)	1.13 (0.06)	0.97 (0.04)
19	202[M+](10) 96(100) 81(85)	1350	0.33 (0.02)	0.36 (0.02)	0.27 (0.02)
20	anastreptene	1370	14.87 (0.13)	14.97 (0.11)	15.01 (0.15)
21	α-ylangene	1373	0.47 (0.04)	0.39 (0.02)	0.42 (0.03)
22	β-elemene	1391	0.69 (0.05)	0.73 (0.04)	0.79 (0.05)
23	α-gurjunene	1419	0.30 (0.02)	0.27 (0.01)	0.32 (0.02)
24	204[M+](25) 105(100) 91(92)	1423	0.39 (0.03)	0.36 (0.02)	0.41 (0.03)
25	(-)-aristolene	1427	0.15 (0.01)	0.12 (0.01)	0.10 (0.01)
26	γ-maaliene	1430	0.29 (0.02)	0.33 (0.02)	0.21 (0.01)
27	α-maaliene	1438	0.30 (0.02)	0.31 (0.02)	0.29 (0.01)
28	alloaromadendrene	1457	3.07 (0.09)	3.01 (0.08)	2.99 (0.10)
29	γ-gurjunene	1463	0.70 (0.05)	0.59 (0.06)	0.67 (0.04)
30	204[M+](13) 105(100) 91(85)	1469	0.13 (0.01)	0.09 (0.01)	0.11 (0.01)
31	204[M+](18) 91(100) 105(92)	1471	0.44 (0.03)	0.37 (0.02)	0.31 (0.02)
32	germacrene D	1474	0.33 (0.02)	0.39 (0.03)	0.27 (0.02)
33	ledene	1476	1.91 (0.04)	1.77 (0.05)	1.87 (0.06)
34	bicyclogermacrene	1488	20.75 (0.15)	21.04 (0.12)	20.97 (0.17)
35	γ-humulene	1493	1.77 (0.04)	1.69 (0.05)	1.72 (0.05)
36	cuparene	1502	1.86 (0.05)	1.78 (0.07)	1.98 (0.07)
37	α-bulnesene	1505	0.87 (0.04)	0.82 (0.05)	0.79 (0.04)
38	valencene	1510	0.27 (0.02)	0.22 (0.01)	0.19 (0.02)
39	4,5,9,10-dehydro-isolongifolene	1544	0.27 (0.02)	0.25 (0.02)	0.21 (0.02)
40	218[M+](5) 173(100) 145(97)	1555	0.15 (0.01)	0.11 (0.01)	0.16 (0.01)
41	spathulenol	1570	0.46 (0.03)	0.33 (0.02)	0.37 (0.03)
42	222[M+](5) 107(100) 147(75)	1573	0.37 (0.02)	0.33 (0.02)	0.29 (0.02)
43	220[M+](2) 159(100) 96(75)	1576	0.27 (0.01)	0.22 (0.02)	0.19 (0.01)
44	globulol	1590	-	-	-
45	220[M+](2) 120(100) 105(55)	1593	2.07 (0.08)	2.22 (0.09)	2.14 (0.10)
46	bisabola-2,10-diene 1,9-oxide	1602	0.91 (0.05)	0.86 (0.06)	0.67 (0.05)
47	220[M+](6) 119(100) 91(88)	1606	0.27 (0.02)	0.24 (0.02)	0.18 (0.01)
48	220[M+](2) 94(100) 109(85)	1613	0.87 (0.04)	1.02 (0.05)	0.99 (0.04)
49	ledene oxide-(II)	1629	0.84 (0.05)	0.96 (0.04)	0.78 (0.04)
50	isospathulenol	1631	1.12 (0.07)	1.05 (0.05)	0.97 (0.05)
51	α-acorenol	1633	0.37 (0.02)	0.33 (0.02)	0.24 (0.03)
52	234[M+](2) 105(100) 91(96)	1664	0.41 (0.03)	0.35 (0.03)	0.32 (0.03)
53	234[M+](3) 109(100) 91(85)	1672	0.34 (0.02)	0.47 (0.05)	0.57 (0.04)
54	aromadendrane-4,10-diol	1683	0.97 (0.05)	1.73 (0.06)	1.54 (0.05)

55	234[M+](4) 41(100) 109(98)	1686	0.39 (0.03)	0.42 (0.03)	0.36 (0.02)
56	232[M+](1) 105(100) 159(60)	1691	-	-	-
57	236[M+](1) 43(100) 139(65)	1694	29.57 (0.18)	27.98 (0.21)	29.61 (0.25)
58	1,4-dimethyl-7-isopropyl-azulene	1772	0.81 (0.04)	0.73 (0.05)	0.69 (0.04)
59	276[M+](1) 173(100) 109(50)	1805	-	-	-
60	276[M+] (8) 95(100) 43(66)	1818	-	-	-
61	280[M+](1) 110(100) 91(50)	1849	2.37 (0.06)	2.57 (0.04)	2.41 (0.07)
62	280[M+](1) 147(100) 119(70)	1924	0.11 (0.01)	0.09 (0.01)	0.10 (0.01)
63	geranyllinalool	2034	0.11 (0.01)	0.18 (0.01)	0.19 (0.02)
64	280[M+](2) 91(100) 105(85)	2041	0.12 (0.01)	0.14 (0.01)	0.15 (0.01)
65	280[M+](1) 91(100) 77(59)	2063	-	-	-
	Total		99.53 (2.05)	98.68 (2.08)	98.93 (2.17)
	% Identified		60.63 (1.42)	61.05 (1.40)	60.14 (1.45)
	Including the following:				
	Aliphatics		0.79 (0.04)	0.74 (0.05)	0.70 (0.04)
	Aromatics		3.32 (0.13)	3.38 (0.15)	3.28 (0.13)
	Monoterpene hydrocarbons		0.97 (0.07)	0.95 (0.06)	0.94 (0.06)
	Sesquiterpene hydrocarbons		50.77 (0.90)	50.54 (0.88)	50.46 (0.95)
	Sesquiterpenoide hydrocarbons		4.78 (0.28)	5.44 (0.26)	4.76 (0.27)

- less than 0.01%. [a] Retention index on Quadrex 007-5MS column. [b] For abbreviations of samples see Table S10. Mean % and ( ) standard deviation for sample analyzed in triplicate.

Table S3. Chemical Abstracts Service registry number (CAS) and methods used to identify compounds, including volatile compounds, detected in samples of *C. sphagnicola* f. *sphagnicola* (CSS), *C. sphagnicola* f. *paludosa* (CSP) and *C. sphagnicola* LC (CSL).

No.	Compounds	RI [a]	Methods of compounds identities [b]	CAS
1	3-hydroxybutan-2-one	<700	1, 2, 3, 5	513-86-0
2	3-methylbutan-1-ol	706	1, 2, 3, 5	123-51-3
3	hexan-1-ol	867	1, 2, 3, 5	111-27-3
4	tricyclene	926	2, 3, 4, 5	508-32-7
5	α-pinene	936	1, 2, 3, 4, 5	2437-95-8
6	2-methylpentan-2,4-diol	938	1, 2, 3	107-41-5
7	camphene	953	1, 2, 3, 4, 5	79-92-5
8	benzaldehyde	960	1, 2, 3, 4, 5	100-52-7
9	β-pinene	978	1, 2, 3, 4, 5	127-91-3
10	7-octen-4-ol	982	2, 3, 5	53907-72-5
11	3-carene	1009	1, 2, 3, 4, 5	13466-78-9
12	benzenemethanol	1023	1, 2, 3, 4	100-51-6
13	120[M+](16) 91(100) 79(35)	1041		
14	benzeneethanol	1114	2, 3, 4, 5	60-12-8
15	β-cyclocitral	1222	1, 2, 3, 4, 5	432-25-7
16	phenoxyethanol	1226	1, 2, 3, 5	122-99-6
17	189[M+](3) 121(100) 93(90)	1322		
18	bicycloelemene	1325	2, 3, 5	32531-56-9
19	202[M+](10) 96(100) 81(85)	1350		
20	anastreptene	1370	1, 2, 3, 6	64340-33-6
21	α-ylangene	1373	2, 3, 5	14912-44-8
22	β-elemene	1391	2, 3, 4	33880-83-0
23	α-gurjunene	1419	2, 3, 4, 5	489-40-7
24	204[M+](25) 105(100) 91(92)	1423		
25	(-)-aristolene	1427	2, 3, 5	6831-16-9
26	γ-maaliene	1430	2, 3, 5	20071-49-2
27	α-maaliene	1438	5, 6	489-28-1
28	alloaromadendrene	1457	2, 3, 5	25246-27-9
29	γ-gurjunene	1463	2, 3, 4, 5	22567-17-5
30	204[M+](13) 105(100) 91(85)	1469		
31	204[M+](18) 91(100) 105(92)	1471		
32	germacrene D	1474	2, 3, 4, 5	23986-74-5
33	ledene	1476	2, 3	21747-46-6
34	bicyclogermacrene	1488	1, 2, 3, 4, 5	67650-90-2
35	γ-humulene	1493	2, 3, 5	26259-79-0
36	cuparene	1502	1, 2, 3, 4, 5	16982-00-6
37	α-bulnesene	1505	2, 3, 4, 5	3691-11-0
38	valencene	1510	1, 2, 3, 4, 5	4630-07-3
39	4,5,9,10-dehydro-isolongifo-lene	1544	2, 3	156747-45-4
40	218[M+](5) 173(100) 145(97)	1555		
41	spathulenol	1570	2, 3, 4, 5	6750-60-3
42	222[M+](5) 107(100) 147(75)	1573		
43	220[M+](2) 159(100) 96(75)	1576		
44	globulol	1590	2, 3, 4, 5	51371-47-2
45	220[M+](2) 120(100) 105(55)	1593		
46	bisabola-2,10-diene 1,9-oxide	1602	2, 3	
47	220[M+](6) 119(100) 91(88)	1606		
48	220[M+](2) 94(100) 109(85)	1613		
49	ledene oxide-(II)	1629	2, 3, 5	
50	isospathulenol	1631	2, 3, 5	88395-46-4

51	α-acorenol	1633	2, 3, 4, 5	28296-85-7
52	234[M+](2) 105(100) 91(96)	1664		
53	234[M+](3) 109(100) 91(85)	1672		
54	aromadendrane-4,10-diol	1683	2, 3, 5	70051-38-6
55	234[M+](4) 41(100) 109(98)	1686		
56	232[M+](1) 105(100) 159(60)	1691		
57	236[M+](1) 43(100) 139(65)	1694		
58	1,4-dimethyl-7-isopropyl-azulene	1772	2, 3, 4	489-84-9
59	276[M+](1) 173(100) 109(50)	1805		
60	276[M+] (8) 95(100) 43(66)	1818		
61	280[M+](1) 110(100) 91(50)	1849		
62	280[M+](1) 147(100) 119(70)	1924		
63	geranyllinalool	2034	1, 2, 3, 4, 5	1113-21-9
64	280[M+](2) 91(100) 105(85)	2041		
65	280[M+](1) 91(100) 77(59)	2063		

[a] Retention index on Quadrex 007-5MS column; [b] Methods of compounds identities: 1 - reference standards; 2 - NIST; 3 - NIST Chemistry WebBook; 4 - Adams 4 Library; 5 – Pherobase; 6 - literature data

Table S4. One-way ANOVA and post-hoc Scheffé test showing statistically significant differences between genetic groups of *C. sphagnicola*: *C. sphagnicola* f. *sphagnicola* (CSS), *C. sphagnicola* f. *paludosa* (CSP), and *C. sphagnicola* LC (CSL). Table shows p values.

Compounds	All groups	post hoc Scheffé test		
		CSS-CSP	CSS-CSL	CSP-CSL
3-hydroxy-2-butanone	0,00000	0,000000	0,006095	0,018362
3-methyl-1-butanol	0,00000	0,000140	0,009154	0,000001
1-hexanol	0,00000	0,000004	0,001065	1,000000
tricyclene	0,00000	0,000000	0,003107	0,010337
α -pinene	0,00000	0,000140	0,011601	0,000001
2-methyl-2,4-pentandiol	0,00000	0,000004	0,001065	1,000000
camphene	0,00000	0,000431	0,040581	0,000002
benzaldehyde	0,00000	0,000000	0,018362	0,760946
β-pinene	0,00000	0,000140	0,010134	0,000001
7-octen-4-ol	0,00000	0,000004	0,001065	1,000000
3-carene	0,00000	0,000004	1,000000	0,001065
benzenemethanol	0,00000	0,000145	0,884543	0,000154
120[M+](16) 91(100) 79(35)	0,00060	1,000000	0,001657	0,000676
benzeneethanol	0,00000	0,000106	0,017413	0,000002
β-cyclocitral	0,00050	1,000000	0,000487	0,002241
phenoxyethanol	0,00000	0,000140	0,000001	0,016243
189[M+](3) 121(100) 93(90)	0,00000	0,000060	0,000008	0,036288
bicycloelemene	0,00000	0,000312	0,000002	0,011712
202[M+](10) 96(100) 81(85)	0,00000	0,000140	0,000003	0,000001
anastreptene	0,00000	0,000140	0,000001	0,045116
α-ylangene	0,00000	0,000140	0,000226	1,000000
β-elemene	0,00020	0,195014	0,018362	0,000122
α-gurjunene	0,00000	0,000312	0,000002	0,037509
204[M+](25) 105(100) 91(92)	0,00000	0,000005	0,000787	1,000000
aristolene	0,00000	0,002893	0,000009	0,048776
γ-maaliene	0,00000	0,000140	0,000001	0,038624
α-maaliene	0,00020	0,248820	0,000090	0,011412
alloaromadendrene	0,00000	0,045334	0,021040	0,000026
γ-gurjunene	0,00000	0,000140	0,000001	0,006410
204[M+](13) 105(100) 91(85)	0,00000	0,000089	0,022148	0,000003
204[M+](18) 91(100) 105(92)	0,00000	0,000060	0,036288	0,000008
germacrene D	0,00000	0,000445	0,022530	0,000014
ledene	0,00000	0,000075	0,000005	0,027888
bicyclogermacrene	0,00000	0,000140	0,010162	0,000001
γ-humulene	0,00000	0,000000	0,046659	0,023212
cuparene	0,00000	0,000000	0,018286	0,048160
α-bulnesene	0,00000	0,000490	0,000002	0,047931
valencene	0,00000	0,000000	0,032108	0,111659
4,5,9,10-dehydro-isolongifolene	0,00020	0,240467	0,011627	0,000087
218[M+](5) 173(100) 145(97)	0,00000	0,000239	0,000001	0,010337
spathulenol	0,00000	0,000140	0,000001	0,024344
222[M+](5) 107(100) 147(75)	0,00000	0,000140	0,000001	0,001243
220[M+](2) 159(100) 96(75)	0,00000	0,000140	0,000001	0,001160
globulol	0,00000	0,000140	0,000001	0,003117
220[M+](2) 120(100) 105(55)	0,00000	0,000140	0,000001	0,016243
bisabola-2,10-diene 1,9-oxide	0,00000	0,000000	0,043622	0,036288
220[M+](6) 119(100) 91(88)	0,00000	0,000140	0,000001	0,008145
220[M+](2) 94(100) 109(85)	0,00000	0,000312	0,000002	0,047509
ledene oxide-(II)	0,00000	0,000140	0,000001	0,000001
isospathulenol	0,00000	0,000000	0,000002	0,000093
α-acorenol	0,00000	0,000025	0,000124	1,000000

234[M+](2) 105(100) 91(96)	0,00000	0,000019	1,000000	0,000081
234[M+](3) 109(100) 91(85)	0,00020	0,309000	0,000135	0,012069
aromadendrane-4,10-diol	0,00000	0,000355	0,044685	0,000002
234[M+](4) 41(100) 109(98)	0,00000	0,000000	0,001160	0,043438
232[M+](1) 105(100) 159(60)	0,00000	0,000160	0,000001	0,037512
236[M+](1) 43(100) 139(65)	0,00000	0,000140	0,000001	0,000003
1,4-dimethyl-7-isopropyl-azulene	0,00060	1,000000	0,001971	0,000561
276[M+](1) 173(100) 109(50)	0,00000	0,000140	0,000001	0,000611
276[M+] (8) 95(100) 43(66)	0,00000	0,000973	0,000003	0,044516
280[M+](1) 110(100) 91(50)	0,00000	0,000292	0,048948	0,000002
280[M+](1) 147(100) 119(70)	0,00000	0,000028	0,000119	1,000000
geranyllinalool	0,00000	0,000002	1,000000	0,008004
280[M+](2) 91(100) 105(85)	0,00000	0,000000	0,014999	0,049562
280[M+](1) 91(100) 77(59)	0,00000	0,000004	0,001065	1,000000

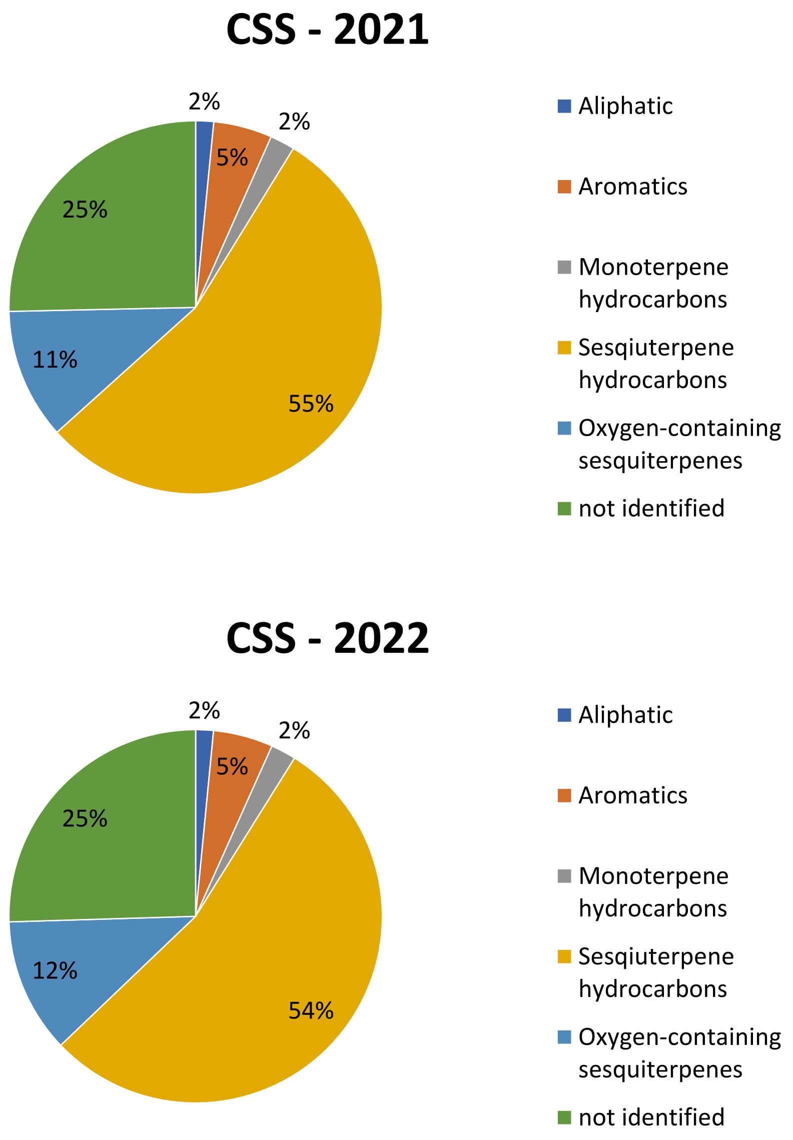

Figure S2. (a) Pie charts showing groups of chemical compounds detected in the *C. sphagnicola* f. *sphagnicola* (CSS) samples collected in 2021 and 2022.

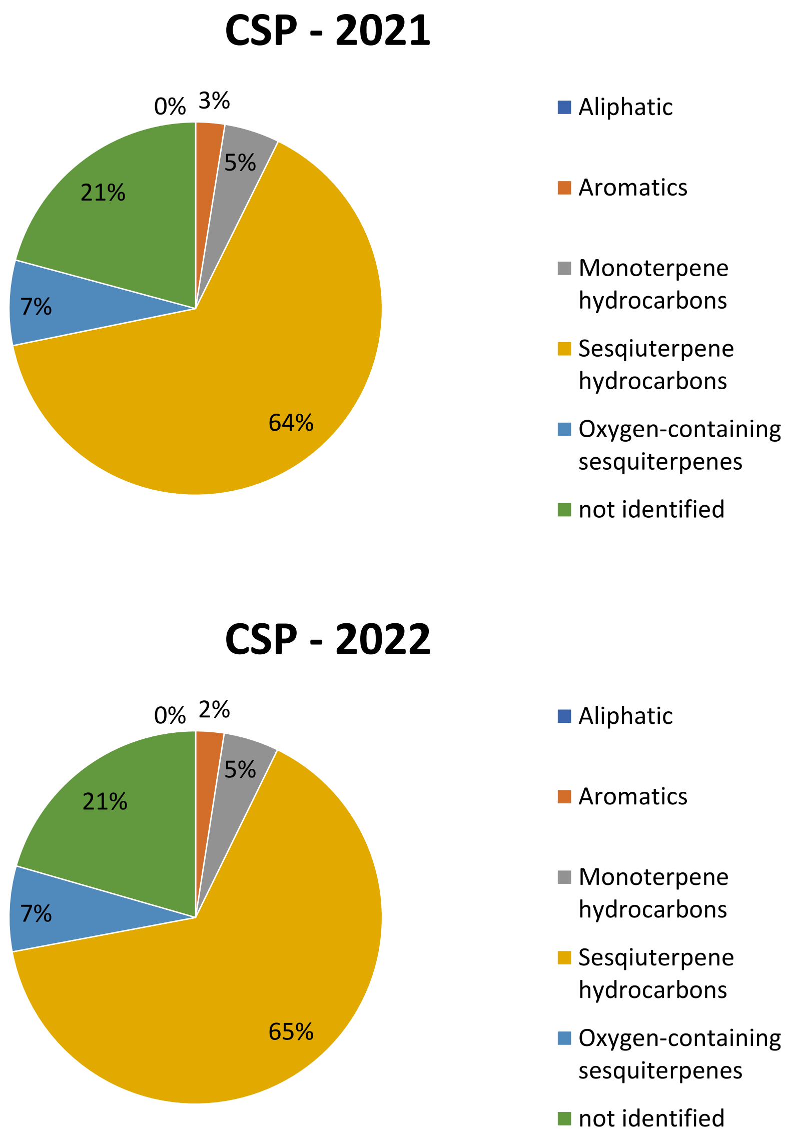

Figure S2. (b) Pie charts showing groups of chemical compounds detected in the *C. sphagnicola* f. *palludosa* (CSP) samples collected in 2021 and 2022.

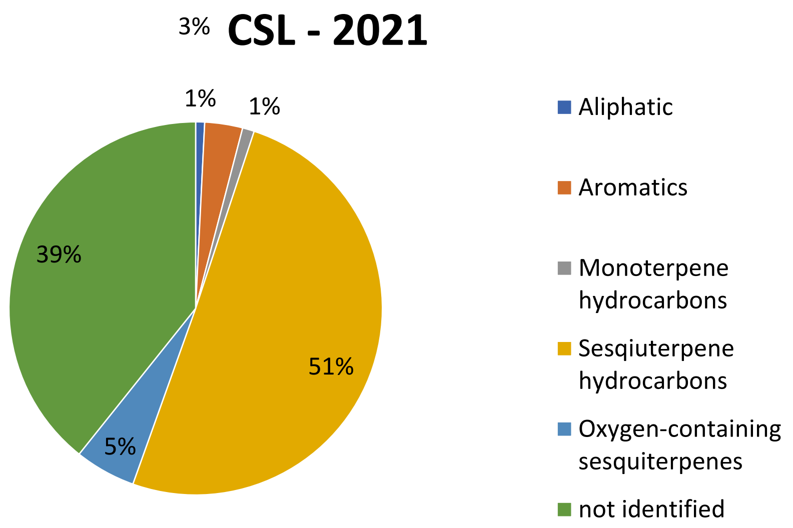

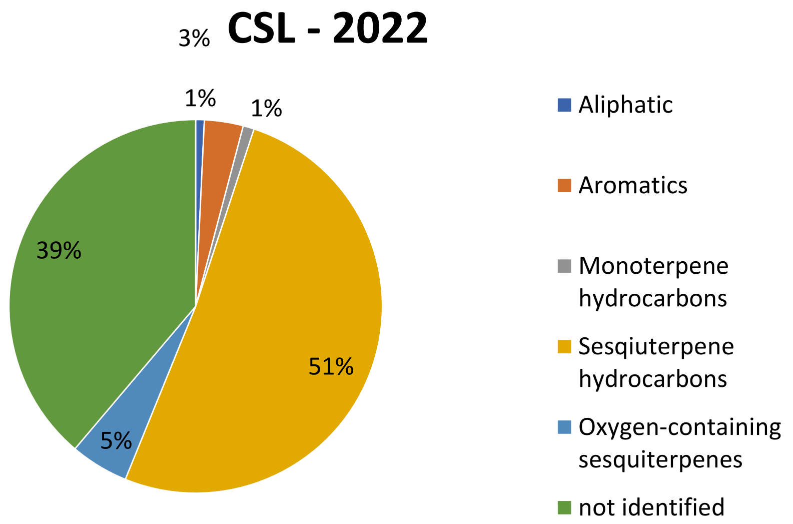

Figure S2. (c) Pie charts showing groups of chemical compounds detected in the *C. sphagnicola* LC samples (CSL) collected in 2021 and 2022.

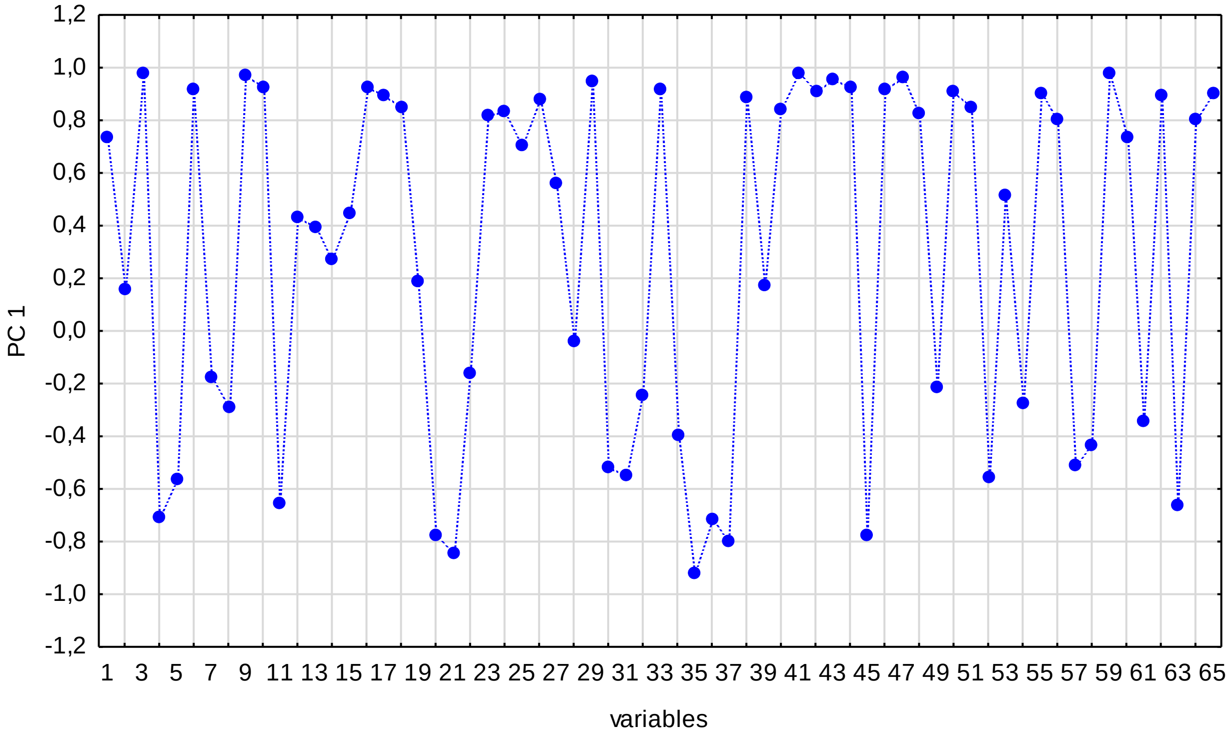

Figure S3. (a) Linear plot of the factor lodgings for the first principal component PC1 based on all the detected VOCs in the *C. sphagnicola* samples.

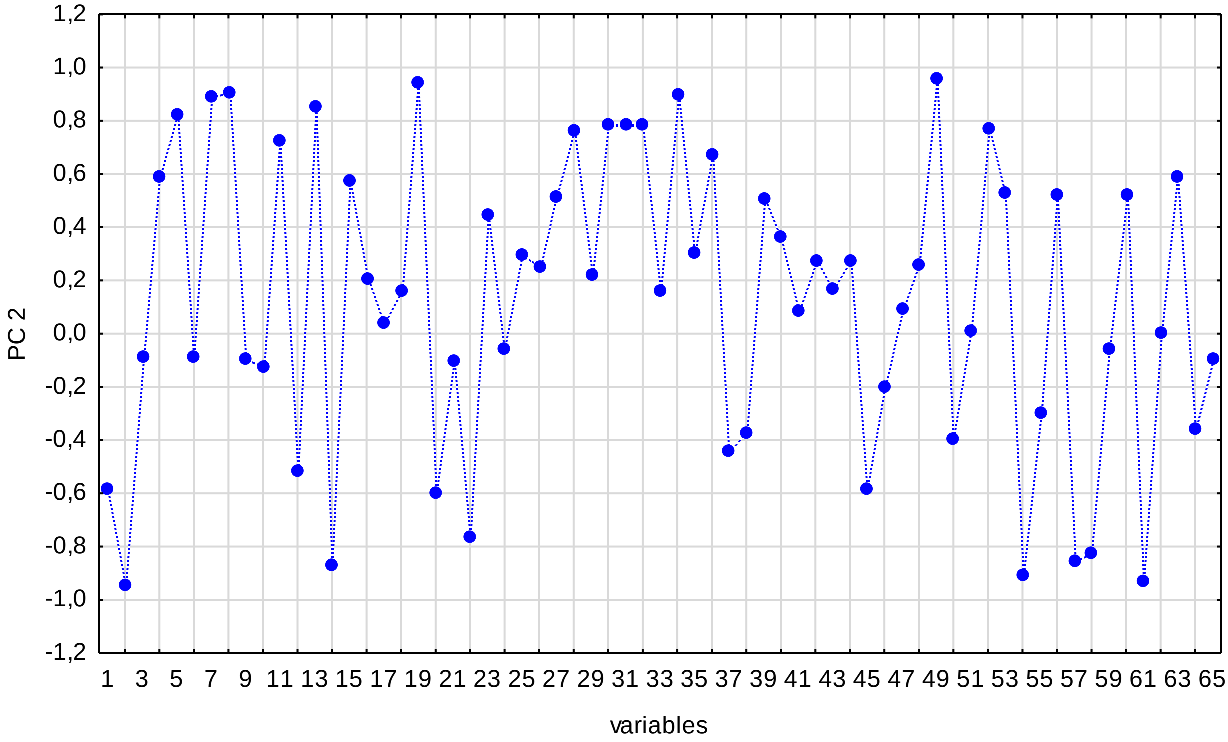

Figure S3. (b) Linear plot of the lodgings for the second principal component PC2 based on all the detected VOCs in the *C. sphagnicola* samples.

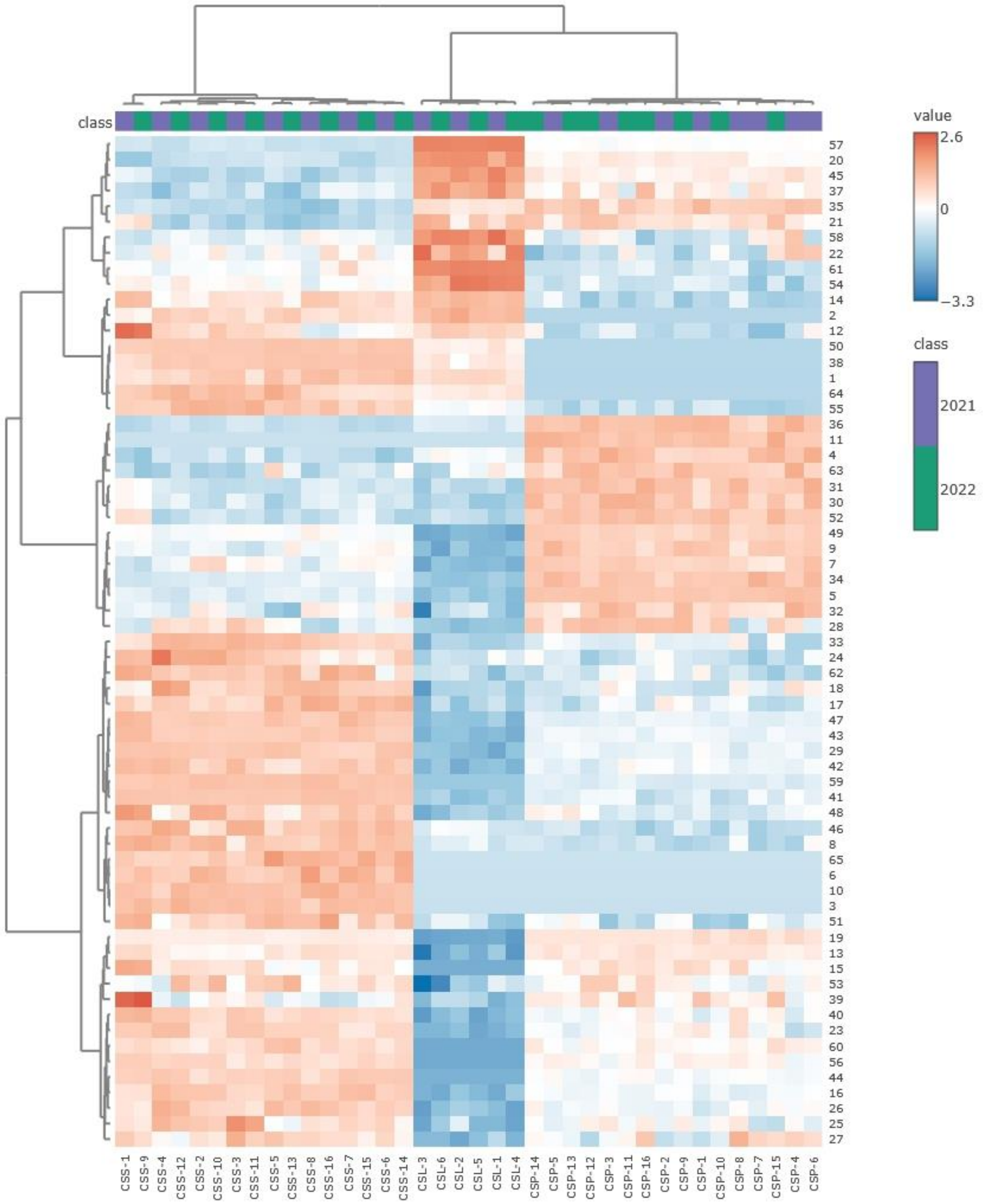

Figure S4. Heatmap clustering of VOC profiles from *C. sphagnicola* samples: *C. sphagnicola* f. *sphagnicola* (CSS), *C. sphagnicola* f. *paludosa* (CSP), and *C. sphagnicola* LC (CSL). The annotations bar shows the clustering of the samples by collection year (class). Each cell was colored based on the level of the concentration of the chemical compound in the sample.

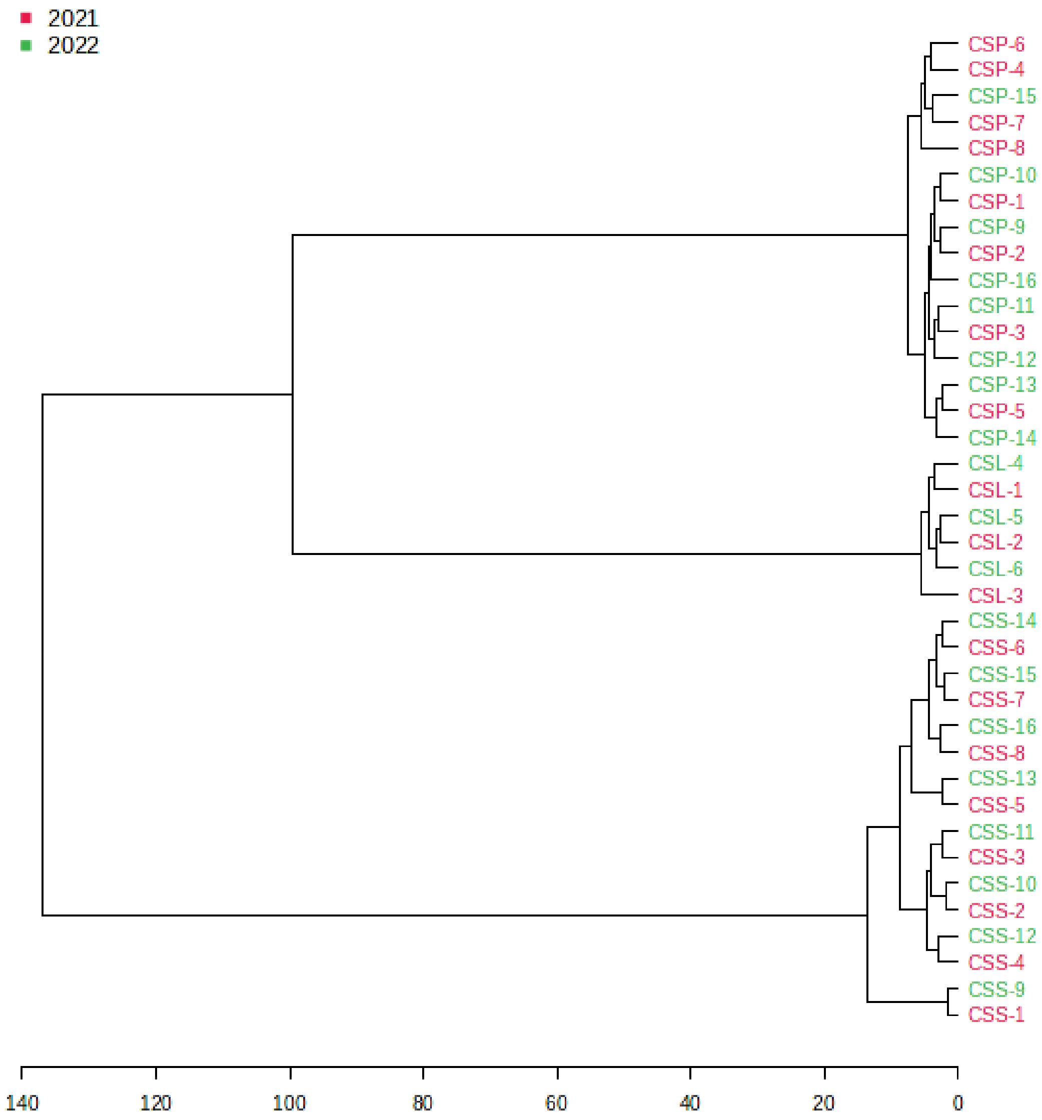

Figure S5. Dendrogram showing the results of the hierarchical cluster analysis of *C. sphagnicola* samples (*C. sphagnicola* f. *sphagnicola* (CSS), *C. sphagnicola* f. *paludosa* (CSP), and *C. sphagnicola* LC (CSL)) constructed based on the Euclidean distance and Ward's linkage method using all the detected VOCs in the samples collected in 2021 and 2022.

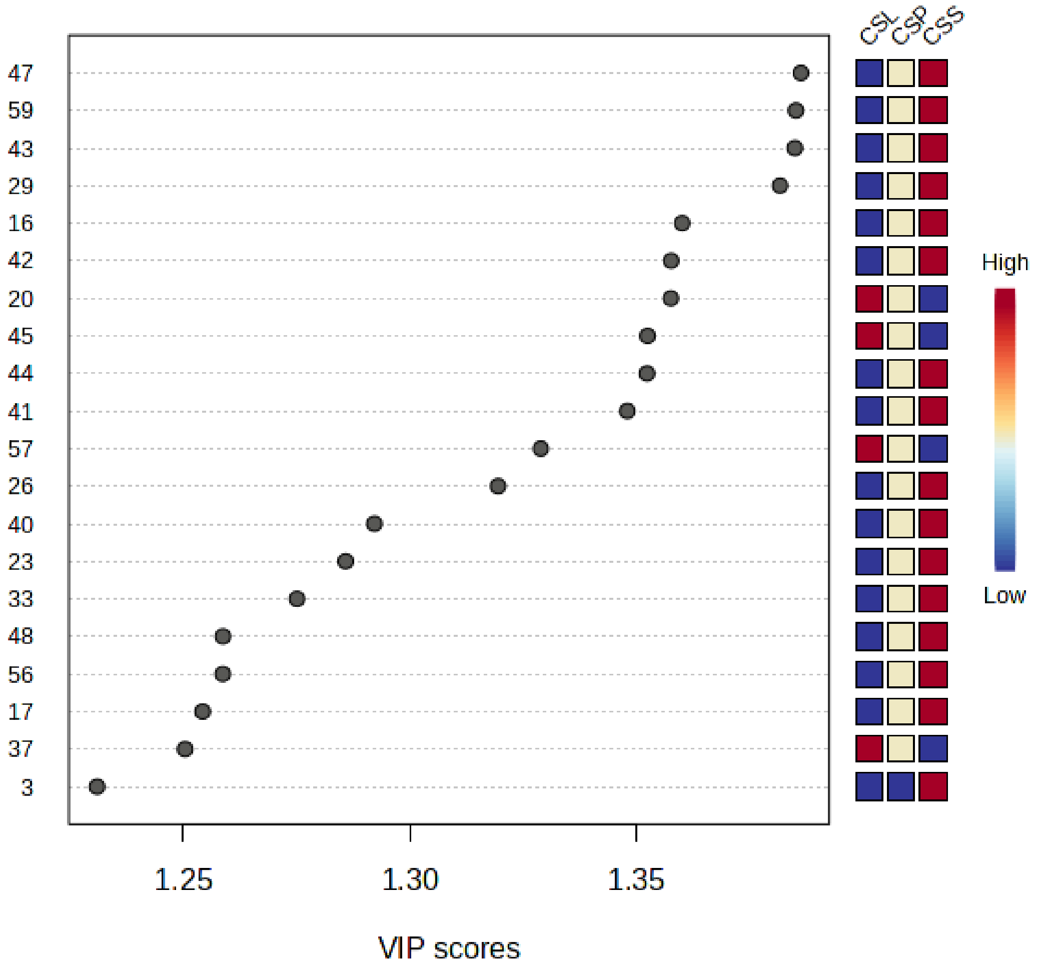

Figure S6. Variable importance in projection (VIP) identified by PLS-DA. The red and blue boxes on the right indicate whether the compound concentration was increased (red) or decreased (blue) in the samples of the three studied groups of *C. sphagnicola*: *C. sphagnicola* f. *sphagnicola* (CSS), *C. sphagnicola* f. *paludosa* (CSP), and *C. sphagnicola* LC (CSL).

Table S5. The *C. sphagnicola* f. *sphagnicola* sampling data in the 2021 year used for the study.

Sample Code	Collection Place	Geographical Coordinates	Date month year
CSS-1	North-Eastern Poland, Warmia, Lake Godle	53°53'04.1"N 22°26'31.3"E	08.2021
CSS-2	North-Western Poland, Pomerania, Lake Duże Sitno	54°16'26.1"N 17°30'34.8"E	06.2021
CSS-3	North-Western Poland, Pomerania, Lake Wałachy	54°04'16.0"N 17°56'07.0"E	06.2021
CSS-4	North-Western Poland, Pomerania, Lake Małe Katarzynki	53°56'29.3"N 17°17'51.0"E	06.2021
CSS-5	North-Western Poland, Pomerania, Lake Chądzie	54°00'05.6"N 17°59'49.8"E	06.2021
CSS-6	North-Western Poland, Pomerania, Krwawe Doły	53°59'34.0"N 18°00'31.4"E	06.2021
CSS-7	North-Western Poland, Pomerania, Lake Czarne	54°22′50.7″N 18°12′07.6″E	06.2021
CSS-8	North-Western Poland, Pomerania, Lake Małe Oczko	54°03'23.3"N 18°00'16.4"E	06.2021

Table S6. The *C. sphagnicola* f. *paludosa* sampling data in the 2021 year used for the study.

Sample Code	Collection Place	Geographical Coordinates	Date month year
CSP-1	Southern Poland, Tatra Mts, Żółta Turnia Mts	49°14'03.2"N 20°01'45.5"E	08.2021
CSP-2	Southern Poland, Tatra Mts, Lake Toporowy Wyżni	49°16'46.4"N 20°01'47.7"E	09.2021
CSP-3	Southern Poland, Tatra Mts, Biały Potok glade	49°16'49.0"N 19°51'09.9"E	09.2021
CSP-4	Southern Poland, Tatra Mts, Pańszczyca Valley	49°14'26.8"N 20°02'04.5"E	09.2021
CSP-5	Southern Poland, Tatra Mts, Wielka Pańszczycka Młaka	49°16′07.5″N 20°02′37.0″E	09.2021
CSP-6	Southern Poland, Tatra Mts, Smreczyński Staw pond	49°13′21.7″N 19°51′51.2″E	09.2021
CSP-7	Southern Poland, Tatra Mts, Ornak Mts	49°13′13.0″N 19°50′08.4″E	08.2021
CSP-8	Southern Poland, Tatra Mts, Goryczkowy Wierch Mts.	49°13′51.1″N 19°57′55.8″E	08.2021

Table S7. The *C. sphagnicola* LC sampling data in the 2021 year used for the study.

Sample Code	Collection Place	Geographical Coordinates	Date month year
CSL-1	Southern Poland, Tatra Mts, forest Capowski	49°17'21.0"N 20°02'15.4"E	08.2021
CSL-2	Southern Poland, Tatra Mts, forest Capowski	49°17'35.0"N 20°02'33.2"E	08.2021
CSL-3	Southern Poland, Tatra Mts, forest Capowski	49°17′47,1″N 20°03′15,3″E	09.2021

Table S8. The *C. sphagnicola* f. *sphagnicola* sampling data in the 2022 year used for the study.

Sample Code	Collection Place	Geographical Coordinates	Date month year
CSS-9	North-Eastern Poland, Warmia, Lake Godle	53°53'04.1"N 22°26'31.3"E	08.2022
CSS-10	North-Western Poland, Pomerania, Lake Duże Sitno	54°16'26.1"N 17°30'34.8"E	06.2022
CSS-11	North-Western Poland, Pomerania, Lake Wałachy	54°04'16.0"N 17°56'07.0"E	06.2022
CSS-12	North-Western Poland, Pomerania, Lake Małe Katarzynki	53°56'29.3"N 17°17'51.0"E	06.2022
CSS-13	North-Western Poland, Pomerania, Lake Chądzie	54°00'05.6"N 17°59'49.8"E	06.2022
CSS-14	North-Western Poland, Pomerania, Krwawe Doły	53°59'34.0"N 18°00'31.4"E	06.2022
CSS-15	North-Western Poland, Pomerania, Lake Czarne	54°22′50.7″N 18°12′07.6″E	06.2022
CSS-16	North-Western Poland, Pomerania, Lake Małe Oczko	54°03'23.3"N 18°00'16.4"E	06.2022

Table S9. The *C. sphagnicola* f. *paludosa* sampling data in the 2022 year used for the study.

Sample Code	Collection Place	Geographical Coordinates	Date month year
CSP-9	Southern Poland, Tatra Mts, Żółta Turnia Mts	49°14'03.2"N 20°01'45.5"E	08.2022
CSP-10	Southern Poland, Tatra Mts, Lake Toporowy Wyżni	49°16'46.4"N 20°01'47.7"E	09.2022
CSP-11	Southern Poland, Tatra Mts, Biały Potok glade	49°16'49.0"N 19°51'09.9"E	09.2022
CSP-12	Southern Poland, Tatra Mts, Pańszczyca Valley	49°14'26.8"N 20°02'04.5"E	09.2022
CSP-13	Southern Poland, Tatra Mts, Wielka Pańszczycka Młaka	49°16′07.5″N 20°02′37.0″E	09.2022
CSP-14	Southern Poland, Tatra Mts, Smreczyński Staw pond	49°13′21.7″N 19°51′51.2″E	09.2022
CSP-15	Southern Poland, Tatra Mts, Ornak Mts	49°13′13.0″N 19°50′08.4″E	08.2022
CSP-16	Southern Poland, Tatra Mts, Goryczkowy Wierch Mts.	49°13′51.1″N 19°57′55.8″E	08.2022

Table S10. The *C. sphagnicola* LC sampling data in the 2022 year used for the study.

Sample Code	Collection Place	Geographical Coordinates	Date month year
CSL-4	Southern Poland, Tatra Mts, forest Capowski	49°17'21.0"N 20°02'15.4"E	08.2022
CSL-5	Southern Poland, Tatra Mts, forest Capowski	49°17'35.0"N 20°02'33.2"E	08.2022
CSL-6	Southern Poland, Tatra Mts, forest Capowski	49°17′47,1″N 20°03′15,3″E	09.2022
